# Evidence integration on health damage for humidifier disinfectant exposure and legal presumption of causation

**DOI:** 10.4178/epih.e2023095

**Published:** 2023-10-24

**Authors:** Mina Ha, Taehyun Park, Jong-Hyun Lee, Younghee Kim, Jungyun Lim, Yong-Wook Baek, Sol Yu, Hyen-Mi Chung, Kyu Hyuck Chung, Hae-Kwan Cheong, Review Committee for the Epidemiological Correlations between Humidifier Disinfectants Exposure and Health Effects

**Affiliations:** 1 Department of Preventive Medicine, Dankook University College of Medicine, Cheonan, Korea; 2 Law School of Kangwon National University, Chuncheon, Korea; 3 EH R&C Co., Incheon, Korea; 4 Humidifier Disinfectant Health Center, Environmental Health Research Department, National Institute of Environmental Research, Incheon, Korea; 5 Sungkyunkwan University School of Pharmacy, Suwon, Korea; 6 Sungkyunkwan University School of Medicine, Suwon, Korea

**Keywords:** Humidifier disinfectants, Presumption of causation, *Epidemiological correlation*, Weight-of-the-evidence approach, Systematic reviews, Evidence integration

## Abstract

**OBJECTIVES:**

Inhalation exposure to humidifier disinfectants has resulted to various types of health damages in Korea. To determine the *epidemiological correlation* necessary for presuming the legal causation, we aimed to develop a method to synthesize the entire evidence.

**METHODS:**

Epidemiological and toxicological studies are systematically reviewed. Target health problems are selected by criteria such as frequent complaints of claimants. Relevant epidemiologic studies are reviewed and the risk of bias and confidence level of the total evidence are evaluated. Toxicological literature reviews are conducted on three lines of evidence including hazard information, animal studies, and mechanistic studies, considering the source-to-exposure-to-outcome continuum. The confidence level of the body of evidence is then translated into the toxicological evidence levels for the causality between humidifier disinfectant exposure and health effects. Finally, the levels of epidemiological and toxicological evidence are synthesized.

**RESULTS:**

Under the Special Act revised in 2020, if the history of exposure and the disease occurred/worsened after exposure were approved, and the *epidemiological correlation* between the exposure and disease was verified, the legal causation is presumed unless the company proves the evidence against it. The *epidemiological correlation* can be verified through epidemiological investigations, health monitoring, cohort investigations and/or toxicological studies. It is not simply as statistical association as understood in judicial precedents, but a general causation established by the evidence as a whole, i.e., through weight-of-the-evidence approach.

**CONCLUSIONS:**

The weight-of-the-evidence approach differs from the conclusive single study approach and this systematic evidence integration can be used in presumption of causation.

## INTRODUCTION

Humidifier disinfectants are used to prevent microbial proliferation and slime formation in humidifier water tanks. They were manufactured and sold in Korea from 1994 to 2011. From 2006, there were reports of an outbreak of acute interstitial pneumonia accompanied by severe respiratory failure among children. In 2008, the medical community requested to the health authorities virus testing and survey; however, they were unable to determine the cause. In April 2011, a university hospital reported six cases of severe acute lung disease in perinatal women, and epidemiological investigation on 16 patients revealed that humidifier disinfectants may have been a potential cause. Following the confirmation of humidifier disinfectant toxicity in animal experiments, the health authorities recalled the humidifier disinfectants from the market in November 2011 and officially declared as the cause of lung injury outbreaks in the following year [1].

In August 2013, the government granted financial support to humidifier disinfectant victims who were experiencing urgent difficulties. In March 2014, the governmental authorities for remediation process were changed from the Ministry of Health and Welfare to the Ministry of Environment, and medical and funeral expenses began to be supported based on the Environmental Health Act. In 2016, manufacturers and sellers of humidifier disinfectants were investigated by prosecutors, and the National Assembly convened the Special Committee for the National Investigation of Humidifier Disinfectants to investigate the facts surrounding the incident. In February 2017, the ‘Special Act on Remedy for Damage Caused by Humidifier Disinfectants’ (hereafter referred to as the ‘Special Act’) was enacted. The Special Act established a special remedy account contributed by the manufacturers and sellers of humidifier disinfectant ingredients and products, which was distinct from the governmental relief finances, and the scope of support and eligible recipients was expanded [2].

In August 2018, according to the first amendment of the Special Act, the recipients of support from the special remedy account were granted with legal victim status, and the government contributions were allocated to augment funds of the special remedy account. In March 2020, in the second revision of the Special Act, the funds from the special remedy account were consolidated with governmental relief fund, and the scope of health damage was expanded to a comprehensive definition of “damage to life or health that occurred or worsened following exposure to humidifier disinfectants”. This indicate that the remedy could be provided more broadly, even for those who were not suffering from specific damages that were established as humidifier disinfectant related diseases, such as Humidifier Disinfectant Lung Injury, asthma, fetal injury, pediatric or adult interstitial lung disease, bronchiectasis, or pneumonia. As a result, the process of examination and determination of the damage was completely reformed such that the victims’ overall health condition before and after exposure to humidifier disinfectants was reviewed. In instances if the history of exposure and the disease occurred/worsened after exposure were approved, and the *epidemiological correlation* between the exposure and disease was verified, the legal causation is presumed unless the company proves the evidence against it. This addresses previous gap in the law where proving “significant probability” that humidifier disinfectant exposure caused the damage was particularly challenging for “non-specific diseases”, thus reduces the burden of proof for victims [3].

Prior to the second revision of the Special Act, only 280 victims were recognized by the government. Following the amendments, 7,870 individuals applied for the damage remedy in September 2023; of which, 5,212 were eligible to receive relief benefits from the government. Individuals who suspect disease occurred/worsened due to humidifier disinfectant exposure can report their damage at any time. This ensures that long-term sequelae are also considered for potential compensation.

However, verifying an *epidemiological correlation* demands rigorous scientific investigation, making it challenging to prove for an individual victim. Thus, its enforcement decree indicates that evidence on *epidemiological correlation* needs to be recognized from epidemiological investigations, health monitoring, cohort investigations and/or toxicology studies conducted by Minister of the Environment directly or by professional research institutions [4].

The National Institute of Environmental Research, the Ministry of Environment has been required to organize a review committee of experts from multiple academic disciplines, and continually publishes the “Review report of *Epidemiological Correlations*”, in which a review and evaluation of various existing sources of scientific evidence [5] are conducted. Basically, the review process must be transparent and thoroughly documented for presuming legal causation. This requires an assessment of relevant scientific evidence based on systematic review and a procedure for “verification of *epidemiological correlations*” in accordance with the Special Act. Therefore, this study aimed to provide a specific guideline for verifying the *epidemiological correlation* between humidifier disinfectant exposure and disease, drawing upon various methodologies related with systematic reviews and evidence integration within the fields of medicine and public health. As used in this paper, “*epidemiological correlation*” is a legal term that is a requirement for the presumption of causation as defined by the Special Act.

## PUBLICATION PROCEDURE OF REVIEW REPORTS ON *EPIDEMIOLOGICAL CORRELATIONS*

Publication of a review report of the *epidemiological correlations* between humidifier disinfectant exposure and disease is one of the responsibilities of the government described in the Special Act supporting legal action of victims [3]. The “Review Committee for the *Epidemiological Correlations* between Humidifier Disinfectant Exposure and Health Effects” (hereafter referred to as the Review Committee), comprised experts and civil servants to perform systematic literature reviews and integrate evidence from relevant studies. The Review Committee is composed of 5 expert committees from the fields of environmental exposure science, clinical medicine, epidemiology, toxicology, and law. At least three experts from the Review Committee participate in the expert committees.

**Figure 1.**
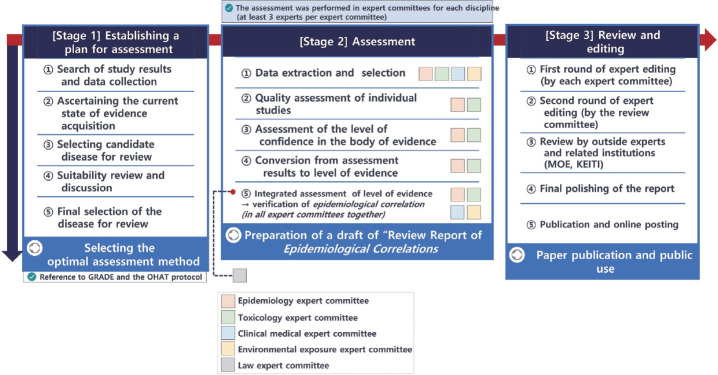
Publication process of the report on *epidemiological correlation* between humidifier disinfectants exposure and diseases. GRADE, Grading of Recommendations, Assessment, Development, and Evaluation; OHAT, Office of Health Assessment and Translation; MOE, Ministry of Environment; KEITI, Korea Environmental Industry & Technology Institute.

The Review Committee assesses whether humidifier disinfectant exposure associates with specific diseases through the review process and determines the final level of evidence. Based on the levels of evidence, the committee decides whether an “*epidemiological correlation*” is verified, which is a key condition for presuming a legally recognized causation (Figure 1).

## SCIENTIFIC EVIDENCE INTEGRATION PROCEDURE FOR VERIFYING *EPIDEMIOLOGICAL CORRELATION*

**Figure 2.**
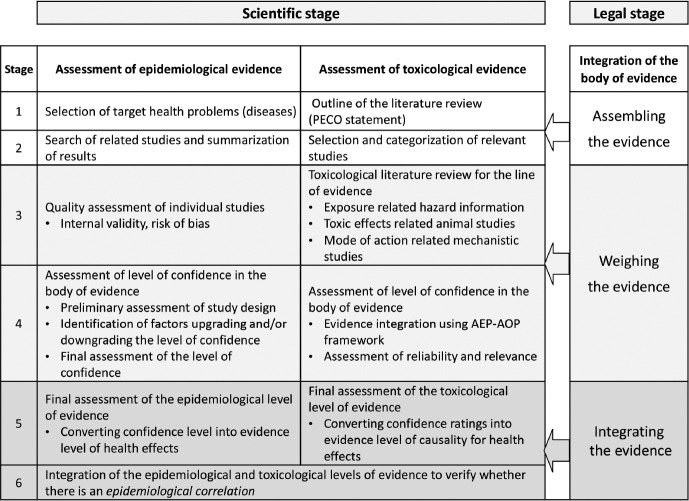
Process to verify the *epidemiological correlation* between humidifier disinfectants exposure and health problems. PECO, population-exposure-comparator-outcome; AEP-AOP, aggregate exposure pathway-adverse outcome pathway.

To verify the *epidemiological correlations* between health problems (disease/illness) and exposure to humidifier disinfectant, based on the weight-of-the-evidence approach, it is important to combine epidemiological evidence with toxicological evidence, such as animal, in vitro, and mechanistic studies. The procedure for verifying *epidemiological correlations* consists of six stages, from selecting the health problems of interest to integrating total evidence from both epidemiology and toxicology (Figure 2).

The method for evidence integration is based on the approach of the Office of Health Assessment and Translation (OHAT), developed by the National Toxicological Program of National Institute of Environmental Health Science, United States, for integrating evidence from systematic literature reviews and risk assessments [6]. We established a new method by adopting this approach to fit our aim of verifying the *epidemiological correlations* between humidifier disinfectant exposure and health problems.

### Assessment and integration of epidemiological evidence

As part of determining the epidemiological association between humidifier disinfectant exposure and health problems, the procedure for assessing the epidemiological level of evidence consists of the following five stages: (1) selection of health problems of interest, (2) search for relevant studies and summarization of their findings, (3) assessment of the quality (internal validity) of individual studies, (4) preliminary assessment of the level of confidence for the body of evidence, identifying of upgrading or downgrading factors, and final assessment of the level of confidence, and (5) final assessment of the epidemiological level of evidence.

#### Selection of health problems of interest

Health problems are selected based on the following considerations: (1) diseases that are often observed among the health damage reporters after humidifier disinfectant use (by analyzing health insurance data of the health damage reporters), (2) diseases with a higher frequency of diagnosis, incidence, or mortality among Korean population during the period of humidifier disinfectant use versus when they were not in sale or banned (by analyzing Nationwide Health Insurance data), (3) diseases frequently complained of by the health damage reporters, (4) diseases frequently detected during health monitoring of victims or individuals confirmed to have been exposed, or (5) other diseases where a review of their *epidemiological correlation* is deemed required.

#### Search for relevant studies and summarization of their findings

In addition to research performed directly by the Minister of Environment, the search includes studies, research reports, regulatory standards, guidelines, and manuals published domestically and internationally in peer-reviewed journals, by domestic or international governmental, regulatory, professional, or research institutions. Keywords are searched in various electronic academic databases, and the retrieved literature is initially filtered based on a review of the titles and abstracts. Duplicate studies, studies where the exposed substance was not humidifier disinfectant, studies not relating to one of the health problems of interest, and papers that were not original studies are excluded. For human studies (epidemiological and clinical) that are initially selected, the full text is reviewed, and the results are summarized into their core content (Supplementary Material 1).

#### Quality assessment for individual studies

Once the selected individual studies have been summarized, the risk of bias is assessed using the method suggested by OHAT. There are several types of bias in epidemiological studies, and their presence is assessed to determine the internal validity or quality of the studies. Since the epidemiological studies on humidifier disinfectant exposure do not include any randomized clinical trials, the categories applicable to randomized clinical trials (e.g., randomization, blinding, double blinding) are excluded [6].

Each bias category is rated on a 4-point scale of “definitely low” (++), “probably low” (+), “probably high” (-), and “definitely high” (--). The focus will be on the core factors of measurement (assessment of exposure and health outcomes) and confounders; while the other factors will also be assessed. Studies with a rating of ++/+ for the core factors and ++/+ for the rest are assigned as Grade 1; those with a rating of -/-- for the core factors and -/-- for the rest are assigned as Grade 3; and the remaining studies are assigned as Grade 2 (Supplementary Material 2).

At least two epidemiologists independently assess the internal validity of the individual studies. Thereafter, a panel of epidemiology experts is formed comprising at least 5 experts, including members in the Expert Committees of Epidemiology as well as outside the Review Committee. During the meeting, the panel reviews the assessment results and works to reach a consensus, which will be the final assessment. Herein, Grade 3 studies are excluded from subsequent evidence integration.

#### Assessment of the level of confidence in the body of evidence for epidemiological studies

An initial confidence rating for body of evidence is determined by study design factors. Subsequently, the final assessment will be performed based on identification of upgrading or downgrading factors. In the initial assessment, one of the four grades (“high”, “medium”, “low”, “inadequate”) will be given based on the following: (1) whether exposure was controlled at experimental level, (2) whether exposure preceded the health outcomes, (3) whether the health outcomes were assessed at individual level, and (4) whether a comparison group exists. Studies with the same design is assigned the same grade in the initial assessment. To integrate the results across the body of evidence, a meta-analysis could be performed if there are a sufficient number of studies; however, meta-analysis is difficult for epidemiological studies relating to humidifier disinfectants. This is attributed to the fact that the definition of exposure vary considerably across the studies, for instances, the time being pre-mandatory or post-mandatory recalls (nationwide cohort), the time of initiating and discontinuing humidifier disinfectant use (damage reporter cohort), indirect indices for the amount of exposure (e.g., total duration of use, mean daily duration of use), or indices estimated directly from the amount of exposure for each individual.

Following a review to check for downgrading factors related to risk of bias, unexplained inconsistency, indirectness, imprecision, and publication bias, as well as upgrading factors related to effect size, dose-response relationship, residual confounding, and consistency between populations, the grades of confidence level can be adjusted [6,7] (Supplementary Material 3). Similar to the initial assessment, four grades can be given corresponding to confidence in whether the apparent relationship between exposure and health effects is likely to be true: high (4+, the relationship is highly likely to be true); moderate (3+, the relationship may be true); low (2+, the relationship may differ from the true effect); and very low (1+ or below, the relationship is highly likely to differ from the true effect).

In cases where several grades were given in the final assessment results, the highest level of confidence is determined as the level of confidence for the body of evidence, regardless of whether a health effect exists or not.

#### Assessment of the epidemiological level of evidence for health problems

The epidemiological body of evidence will be divided based on whether the results exhibit presence or absence of health effects, and the level of confidence in the body of evidence will be translated into an epidemiological level of evidence for health problems. The epidemiological level of evidence either for or against the presence of health effects, will be classified into four levels: “sufficient”, “suggestive”, “unclassifiable”, “unrelated”. In the presence of health effects, if the level of confidence in the body of evidence is “high”, “moderate”, or “low or very low”, the epidemiological level of evidence will be “sufficient”, “suggestive” or “unclassifiable”, respectively. In the absence of health effects, the epidemiological level of evidence will be classified as “unrelated” in cases where the level of confidence in the body of evidence is “high”; otherwise, they will be “unclassifiable” (Table 1).

### Assessment and Integration of toxicological evidence

Collaboration between toxicology and epidemiology is essential to strengthen conclusions regarding the causal relationship between chemical exposure and disease [8]. In this context, a critical role of toxicology is to provide biological plausibility. This provides evidence to determine whether research findings regarding the potential causality between exposure and disease are consistent with existing biological knowledge, particularly in accordance with the principles of toxicology [9].

While toxicology has traditionally been relied on animal studies, the development of non-animal studies using human or animal derived tissues and cells has enabled the use of diverse toxicological data. More recently, the aggregate exposure pathway (AEP) and adverse outcome pathway (AOP) frameworks have been developed to evaluate the source-to-exposure-to-outcome continuum, and these frameworks enhance the human relevance of toxicological studies [10].

The process for assessing the level of toxicological evidence for causality between humidifier disinfectants and health effects includes the following steps: (1) outline of the literature review, (2) selection and categorization of relevant studies, (3) toxicological literature review for the line of evidence, (4) assessment of level of confidence in the body of evidence, and (5) final assessment of the toxicological level of evidence.

#### Outline of the literature review

To assess the level of toxicological evidence regarding the health effects of interest, the scope of the systematic review of the toxicological literature is defined and the population-exposure-comparator-outcome (PECO) framework is described. This approach clearly defines the objectives of the literature review and limits the scope, thereby increasing the validity and reliability of the results of the systematic review.

#### Selection and categorization of relevant studies

Various electronic academic databases will be searched using keywords related to humidifier disinfectants and individual ingredients, diseases associated with health effects, toxic effects, and mode of action, among others. The selected literature will be categorized into three lines of evidence: (1) “hazard information” on the physicochemical characteristics of humidifier disinfectant ingredients, general toxicity, and exposure-related data, (2) “animal studies” that include toxicological research on laboratory animals, and (3) “mechanistic studies” that examine the mode of action in in vitro systems using cells or tissues. Relevant data are extracted from research articles, review articles, and related reports and presentations using the PECO statement.

#### Toxicological literature review for the line of evidence

##### Systematic review of exposure related hazard information

Hazard information is collected from national and international regulatory agencies, including Organization for Economic Cooperation and Development (OECD), European Chemical Agency, and Environmental Protection Agency. This information includes data on physicochemical properties, stability, reactivity, general toxicity, especially inhalation toxicity (single and repeated exposure studies). In addition, data will be extracted from research conducted under conditions that reflect actual use of humidifier disinfectants, including predictions of respiratory deposition and human exposure levels.

A systematic literature review of hazard information will be conducted using the AEP framework [11]. Using all exposure relevant data, an exposure pathway is constructed from the source to the target site where humidifier disinfectants are released into the air and inhaled into the respiratory system.

##### Systematic review of toxic effects related animal studies

For the systematic review of animal studies, relevant literature that indirectly assesses human health effects using laboratory animals such as rats or mice is collected and data on toxic effects, including general toxicity (e.g., weight changes), histopathological or radiological findings, and changes in biomarkers are extracted.

A systematic review of animal studies is performed using the AOP framework to analyze the evidence on toxic effects are analyzed. The reliability of individual studies, including test substances, test animals, study design, test procedures, statistical analyses, test results, and conclusions [12] and the relevance of the adverse outcome to the health effect are evaluated.

##### Systematic review of mode of action related mechanistic studies

For the systematic review of mechanistic studies, relevant literature that investigating the mode of action using human or animalderived cells or tissues is collected and data related to the mode of action, including molecular initiating events, key events, and adverse outcomes, are extracted.

A systematic literature review of mechanistic studies is performed using the AOP framework [13] to analyze the evidence on mode of action. All the data related to the mode of action are used to develop an AOP begins with the initiating reaction between humidifier disinfectants and macromolecules at respiratory target site and progresses through cellular organelles, cells, tissues, and organs, ultimately leading to adverse outcomes.

#### Assessment of level of confidence in the body of evidence

To determine whether humidifier disinfectants are the cause of toxicity associated with health effects, the three lines of evidence (hazard information, animal studies, and mechanistic studies) are integrated into overall body of evidence for the entire source-toexposure-to-outcome continuum using the AEP-AOP framework. The level of confidence in the body of evidence is assessed on the basis of reliability and relevance. Reliability refers to the Hill viewpoints and assesses the consistency between studies in terms of strength and dose-response of key events and adverse outcomes in the AEP-AOP network. Relevance is assessed by referring to the OECD Guidelines for the Evaluation of AOPs [14], evaluating the adequacy of the pathway compared to the hypothesized mechanisms of the health effect, and assessing whether the essentiality of the key events and the sufficiency of the data are supported. The assessment of the confidence level is performed by an expert committee as part of the Review Committee. Categorization includes ‘high’, ‘moderate’, ‘low’, or ‘unclassifiable’ in cases where research is insufficient or ongoing.

#### Final assessment of the toxicological level of evidence

The confidence level of the body of evidence, assessed by integrating the three lines of evidence, is translated to the toxicological level of evidence to determine the causality between humidifier disinfectant exposure and health effects. This is accomplished by integrating the overall body of evidence from hazard information, animal studies, and mechanistic studies on humidifier disinfectants to improve the biological plausibility of the experimental evidence and to strengthen the human relevance of the causal evidence. The level of toxicological evidence is categorized into four levels: “sufficient,” “suggestive,” “unclassifiable,” and “unrelated” (Table 2). If the level of confidence in the body of evidence is “high”, the toxicological level of evidence is “sufficient”; if the level of confidence in the body of evidence is “moderate”, the toxicological level of evidence is “suggestive”; if the level of confidence in the body of evidence is “low”, the toxicological level of evidence is “unrelated”; the level of confidence in the body of evidence is “unclassifiable”, the toxicological level of evidence is “unclassifiable” (Table 2).

### Integration of overall scientific evidence

In accordance with Article 5 of the Special Act, *epidemiological correlation* is interpreted, among legal causations, as a “general causation”, and is assessed by integrating the epidemiological and toxicological levels of evidence for health problems resulting from exposure to humidifier disinfectant.

*Epidemiological correlation* is classified as one of three cases: “verification of an *epidemiological correlation*”, “verification of no *epidemiological correlation*”, or “not determined”. Through evidence integration, “verification of an *epidemiological correlation*” is given when (1) the epidemiological level of evidence is “sufficient”, or (2) the epidemiological level of evidence is “suggestive” but the toxicological level of evidence is “sufficient” or “suggestive”. “Verification of no *epidemiological correlation*” is given in cases where the epidemiological and toxicological levels of evidence are both “unrelated”.

**Figure 3.**
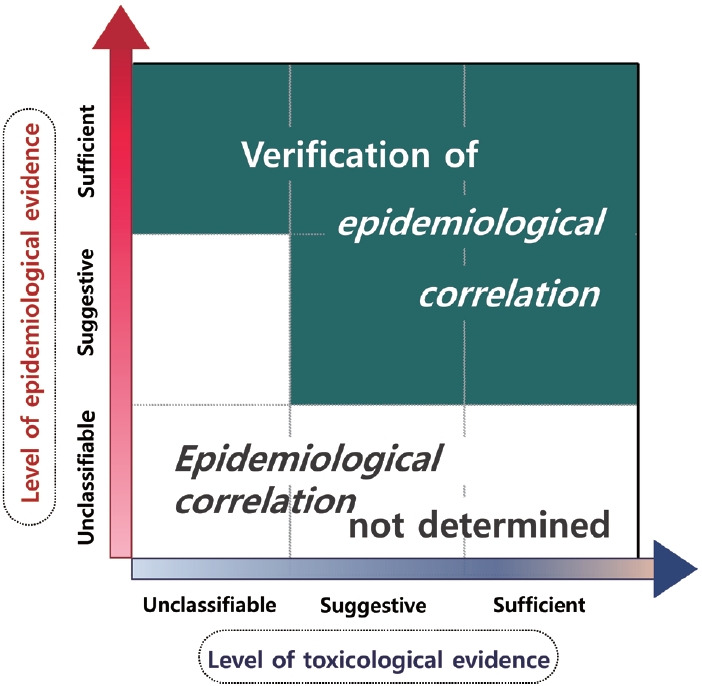
Method to verify the *epidemiological correlation* between humidifier disinfectants exposure and health damages based on the integrated evidence levels of epidemiology and toxicology.

In all other cases, the final classification is “not determined”. However, if the toxicological evidence is “sufficient”, but the type of injury is rare or the incidence of disease is low enough that observational epidemiologic studies are not feasible, and there are clinical cases involving exposure to humidifier disinfectants, the integrated evidence level may be upgraded (Figure 3).

## PROOF OF CAUSATION BY SYSTEMATICALLY EVALUATING AND INTEGRATING SCIENTIFIC EVIDENCE UNDER THE SPECIAL ACT

### Proof of causation based on epidemiological studies

According to the verdict by the Supreme Court of Korea, a causation, as a requirement for establishing a tort, does not need to be indisputably, medically, or scientifically proven, but can be acknowledged even if it is only logically inferred from rule of thumb and a socially accepted idea guided by principle of free evaluation of evidence [15]. The court reduced burden of proof in environmental damages caused by pollution from a “certain” to a “probable” causation. According to the judicial doctrine of probability, if a perpetrator releases a harmful substance that reaches an object that may be harmed, and any loss or harm subsequently occurs, the perpetrator cannot be immune from tort liability unless they prove that the substance was or within safety threshold [16]. In a suit for damage involving physical injury, two stages of causal evidence are required such as general causations and individual causation [16].

Since epidemiology focuses on demonstrating general causations through studies on health and disease in populations, the evidence provided by epidemiological studies is not simply the same as judging causality for individual plaintiffs. If epidemiological studies show a statistical correlation, and there are no confounding factors, such as bias that jeopardize the internal validity of the studies, the resultant association might be inferred to be a general causation. This causal inference is usually made by applying the Hill viewpoints [17]. Of these, only the temporality is essential, while the rest may be satisfied even if it is not a causation, or not be satisfied despite being a causation. However, the likelihood that the epidemiological association is causal increases if more viewpoints are satisfied [17].

Nevertheless, the United States courts have to make a judgment about the causality of damages for an individual plaintiff, and in cases where epidemiological evidence is presented for individual causality, it must not only be sufficiently robust (acceptability), but should also provide sufficient strength of evidence for the potential relationship to be acknowledged for individuals (sufficiency) [18]. However, the Korean court considered that extrapolating causal effects from population-level effect observed or experimented is inadequate for inferring individual causation; for nonspecific diseases that do not show a one-to-one correspondence between cause and outcome, population-level causal effects from epidemiological evidence is not sufficient for proving specific causation for individual plaintiffs [15,16,19,20].

Tetrachlorodibenzodioxin (TCDD)-related injuries in Vietnam, the High Court of Seoul, if epidemiological causality was confirmed, a significant likelihood of individual causality could be acknowledged from the facts of disease occurrence or aggravation after exposure to the harmful substance in individual victims [21]. On the contrary, the Supreme Court ruled that from cause and effect in epidemiological studies, just the potential of disease occurrence could be capable of being inferred [16]. As such, there are differences in proof of causation between specific and nonspecific diseases. For non-specific disease, it is essential to prove not only that the occurrence of a disease is significantly higher in the exposed than the unexposed population, but also to show the likelihood that the non-specific disease was caused by the given risk factor by additionally taking account of information on plaintiffs-related variables (e.g., period and extent of exposure, time of disease onset, health condition before exposure, lifestyle habits, family history, change in disease condition, etc.) (Supplementary Material 4).

### Verification of *epidemiological correlation* in the Special Act

Presumption of causation in Article 5 of the Special Act was satisfied “where it is highly probable that damage to life or health has been caused by a humidifier disinfectant”. However, this was revised in the March 2020 as follows, “where a disease [that] has occurred or worsened after exposure to humidifier disinfectants … [is confirmed to show] an *epidemiological correlation* [with] exposure to humidifier disinfectants”, loosening the burden of proof for victims [3]. Nonetheless, this presumption can be invalidated if the disinfectant supplier proves that “such damage has occurred due to any reason other than the humidifier disinfectants” (Supplementary Material 5).

**Figure 4.**
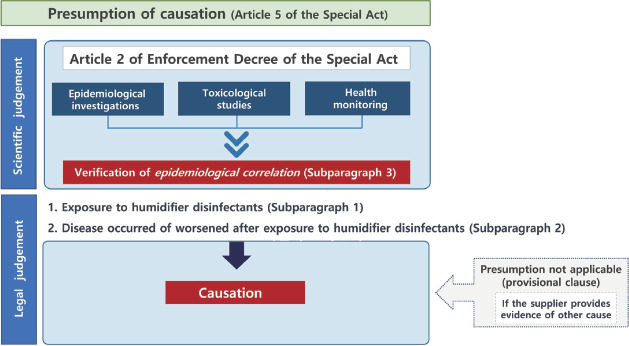
Process of presumption of causation under the Special Act on Remedy for Damage Caused by Humidifier Disinfectants, Article 5.

In summary, under the Special Act, if there is confirmation of (1) the general possibility that humidifier disinfectants can cause or worsen the given disease, (2) the plaintiff’s exposure to the humidifier disinfectant, and (3) the occurrence or aggravation of the disease after the exposure, the presumption of legal causation can be made (provided that the supplier does not offer any counterevidence) (Figure 4).

The revised Act aims to relax the requirements for presumption of causation by taking into account of the lack of relevant information and expert knowledge for proof of causation on the side of victims. In the several revised bills, causation could be presumed simply with the facts of exposure to humidifier disinfectant and the disease occurred or worsened after exposure. The clause allowing the supplier to disprove, that is, the victim’s disease was due to other causes was added in a counterproposal at the National Assembly’s Environment and Labor Committee [22]. At the Legislation and Judiciary Committee, the Ministry of Justice and Office of Court Administration raised concerns that excessive expansion of the scope of presumption of causation could contravene the rule of fault liability; Considering these concerns, the requirement was added that an *epidemiological correlation* between humidifier disinfectant exposure and the disease must be demonstrated [23].

### Proof of causation using a body of evidence approach

According to Article 2 of the Enforcement Decree of the Special Act, finding of *epidemiological correlation* is based not only on epidemiological investigations but also on other available source of evidence, such as health monitoring and toxicology studies (performed either directly by the Minister of Environment or by a professional research institution) [4]. What the Special Act implies are as follows: First, the *epidemiological correlation* should not be understood simply as a statistical association as the same that judicial decisions said. Second, the *epidemiological correlation* must be verified through an integrative review process after evaluating the strength of evidence from various sources of evidence available under the legislation.

The court acknowledges *epidemiological correlation* as a statistical association confirmed in the population and recognizes that it signifies ‘general causation’ when a strong statistical association is presented [16]. However, scientifically, the concept of “*epidemiological correlation*” can have a wide range of relationships, not limited to statistical association, which includes correlation, association, and causation. Moreover, the evidentiary level for *epidemiological correlation* increases from correlation through association to causation (Supplementary Material 6). Thus, the term of *epidemiological correlation* in the Special Act does not solely include cases where indices of association between humidifier disinfectant and disease (i.e., relative risk) show high value, but rather consider various types of relationship and a wide range of evidentiary levels. Various roles of humidifier disinfectant exposure on the occurrence or aggravation of disease need to be understood, not only as the main, direct cause, but also as an indirect, complementary, surrounded environmental, or contributing cause via interactions with other causal factors.

In incidents of damages affecting a group due to exposure to an environmental hazard, such as a chemical substance, it is primarily essential to confirm the association between the exposure of the factor and the health effects at the population level by an epidemiological investigation. Experimental studies like randomized clinical trials, are considered the gold standard for confirming a causal association but are not a feasible option for hazardous substances such as humidifier disinfectants. Moreover, observational epidemiologic studies are difficult to conduct when the disease is rare, the number of observations is too small to achieve statistical power, or the appropriate population or data source for study does not exist. In that case, by considering all available evidence, including exposure characteristics and clinical course and presentation of an affected case or case series, clinical follow-up data such as health monitoring, and toxicological studies from in vitro, mechanistic and animal experiments, the likelihood of adverse health effects from a particular hazard should be determined.

On the other hand, courts understand causation in law as a distinction between “factual” (or natural) causation and “legal” (or ideological) causation (i.e., significant probability), with legal causation presupposing factual causation [24]. However, the degree of proof of factual and natural causation in the legal (trial) realm is determined from a “normative perspective,” as “the review of the existence of factual causation should be limited to the extent necessary to determine the existence of legal causation [25]. A finding of factual causation requires proof by natural or medical evidence. There are two ways to infer factual causation from natural scientific or (clinical) medical evidence: the single evidence approach and the body of evidence approach. In the single evidence approach, the aim is to find a single dispositive study that fully supports a causation, which is commonly adopted by the judges [26]. This approach does not involve any attempt to combine evidence from different studies, and each individual study is reviewed and may be discarded as evidence due to that individual study’s limitations or uncertainty [27]. Conversely, the body of evidence approach assumes that scientific evidence (knowledge) is, by nature, cumulative, and thus aims to derive scientific suggestions from the results of a large number of studies that do not definitely confirm a given conclusion but are suggestive of the conclusion. This approach is typically adopted by scientists and expert administrative offices responsible for environmental and public health issues [28].

This means that the *epidemiological correlation*, which is a major part of the factual (natural) causation determination in the Special Act, must be established through an assessment of the value of the individual evidence, its consistency with the rest of the evidence, and its weight in the totality of the evidence, in accordance with the body of the evidence approach [29] (Supplementary Material 7). *Epidemiological correlation* under the Special Act is a concept that includes, but goes beyond, statistical association as defined in judicial precedent and is established through a comprehensive evaluation and review of the various available evidence. In other words, the evidentiary power of epidemiologic findings may be augmented or supplemented by other investigations and studies such as health monitoring, animal experiments, toxicity studies, etc. The results of such augmentation or complementation, which are derived, interpreted, and evaluated through an integrated review process of all evidence (=synthesis of all evidence), is the process of verifying *epidemiological correlations*.

## DISCUSSION

**Figure 5.**
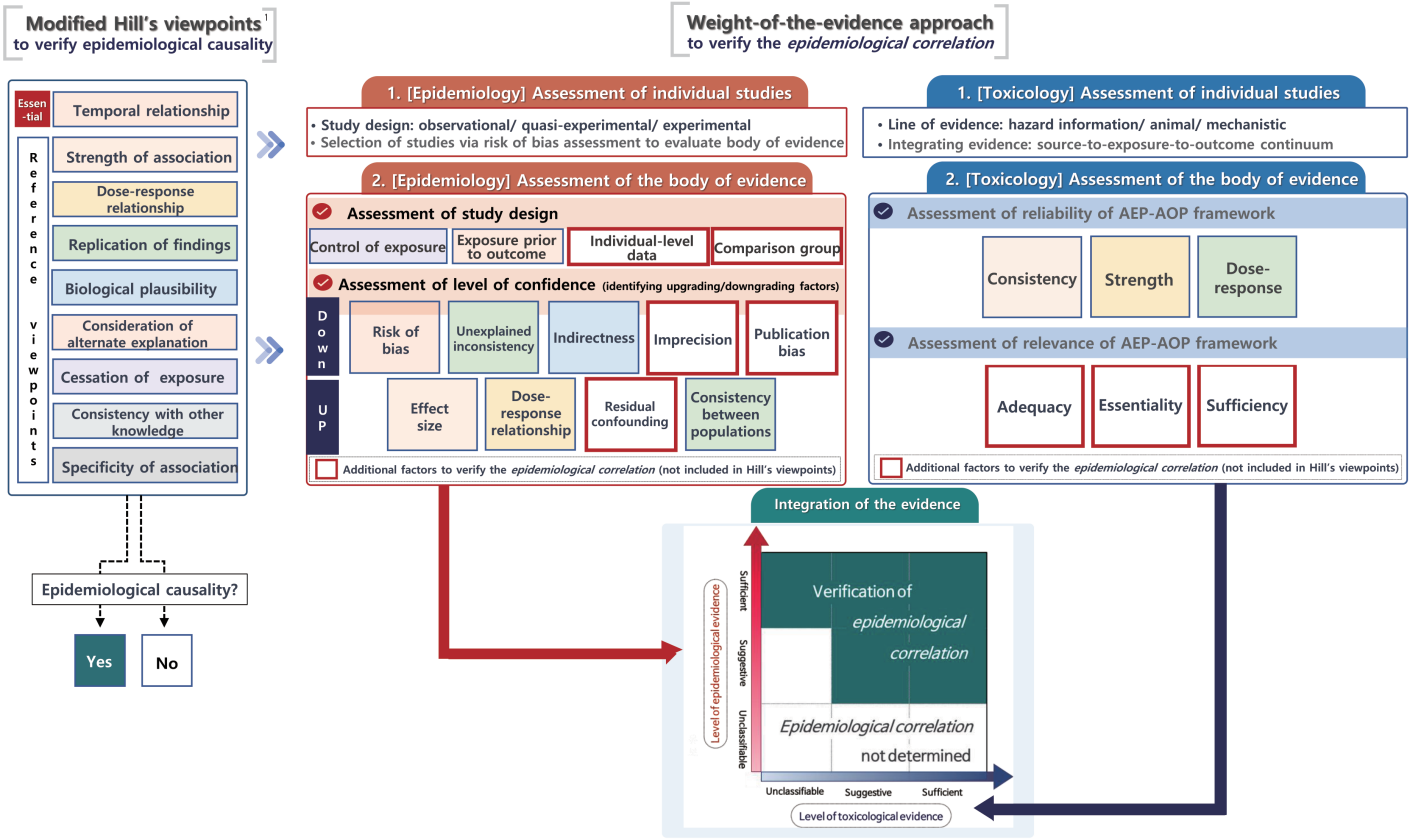
Comparison of weight-of-the-evidence approach with Hill’s viewpoints for causal inference. Same colors are the same components in both approaches. AEP, aggregate exposure pathway; AOP, adverse outcome pathway. 1Source for Hill’s revised view-points: US Department of Health, Education and Welfare. 1964.

In this study, the process of verifying *epidemiological correlation*, a requirement for presumption of legal causation, was refined through a systematic review and synthesis of multidisciplinary evidence from epidemiology, toxicology, clinical medicine, exposure studies, and law. More importantly, we applied the OHAT approach to systematic literature review and evidence integration for risk assessment developed by the U.S. National Toxicology Program, to present a new approach to integrating scientific evidence from epidemiology and toxicology (Figure 5).

The process of verifying “*epidemiological correlation*” in this study applies the Hill’s viewpoints (Supplimentary Material 8) but differes in several ways.

Hill [30] presented the viewpoints for judging whether an observed epidemiological association was causative. Since then, major developments in medicine and biology have vastly improved our understanding of disease causes. The ideas presented by Hill have been continually updated to reflect the latest advances in medicine and biology, and recently, nine viewpoints have been proposed (temporal relationship, strength of the association, dose-response relationship, replication of findings, biologic plausibility, consideration of alternate explanation, cessation of exposure, consistency with other knowledge, and specificity of the association) [31].

Most importantly, these viewpoints should not be judged on an absolute basis. Conclusions about causality can be greatly strengthened when there are multiple forms of evidence supporting that causality from multiple sources. However, it is rare for all of these viewpoints to fit exactly, and it is more likely that one or two viewpoints will be met and a rough pattern of evidence will be judged. Temporal relationship is the only essential viewpoint to demonstrate causality, and the others are caveats [32].

For the dose-response relationship, it is not uncommon to see a threshold dose below which there is no response, or non-linear responses to increasing exposure dose, such as curves of U, S, and J shapes. Here, even if the relationship between cause and outcome is causal, a linear dose-response curve may not be observed for some or all the range of exposed doses. Furthermore, if susceptibility varies by age or sex, or if it is synergistic or antagonistic with other factors, the dose-response relationship may show a completely different pattern [32]. Failure to account for these aspects could lead to incorrect conclusions about causality.

Epidemiological observations can sometimes precede the latest biological knowledge, which can make causations difficult to explain biologically. Hill himself acknowledged that this view of biological probability is limited by the current state of knowledge [17].

Consideration of alternate explanation is another commonly mis-applied viewpoint. This view understands the role of third factors as a confounding effect only and seems to be rooted in a single pathogen-oriented etiology [30]. So-called a single etiology, the ideas that a disease has a single cause, is a concept of the modern Renaissance, when biomedicine took great strides forward, including the invention of the microscope and the discovery of bacteria. Contemporary etiology, which is dominated by chronic diseases, does not believe that any disease is caused by a single factor, even in infectious disease caused by a pathogen [31].

Health and disease are determined by complex interactions between diverse, multi-layered factors, including age, sex, genetics, personal factors such as nutrition and immune function, as well as the physicochemical, social, and institutional environments [31,32]. Each of these factors contribute to pathogenesis through diverse roles at different points on the pathway from exposure to disease. The third factors do not simply have a confounding effect between cause and outcome. The third factor may interact with the factors of interest (the primary exposure) to cause additive, synergistic, or complementary effects, where the presence of third factor allows the factor of interest to contribute to the disease development. Therefore, interpreting Hill’s viewpoint of consideration of alternate explanation as if the role of all factors other than the factor of interest should be ruled out would result in the denial of a true causation. This means that if there are other influential factors other than the factor of interest, the causal factor should not be determined in a confrontational or selective manner, but rather the role, interaction, and contribution of each factor in the entire process of disease development should be considered comprehensively.

“Cessation of exposure” means that a causation can be strongly supported if the disease is significantly reduced or no longer occurs when exposure to the factor of interest is stopped. While the effect of stopping exposure could be observed experimentally, it is possible to observe the effects of a natural or socio-environmental event as if it were an experimental exposure cessation. For instance, after the government ordered a mandatory recall of humidifier disinfectants in November 2011, nationwide investigations conducted by government authorities did not detect a single new case of humidifier disinfectant-associated lung injury defined as humidifier disinfectants lung disease [33]. However, if the disease has a long latent period or the course is irreversible, the disease may develop, persist, or worsen even after stopping exposure. As such, this viewpoint should not be treated as an absolute when inferring causality, and the clinical characteristics and natural course of the disease of interest must be considered.

The “specificity of the association” is suggested to be excluded as viewpoints because no diseases are caused by a single factor [30]. Even in the case of malignant mesothelioma, which is considered an asbestos-induced specific disease, asbestos accounts for 80% of attributable fraction, with the remaining 20% unexplained by asbestos [34].

Currently, it can directly observe and measure detailed incidents and mechanisms in the pathway from exposure to disease that were unknown in the past [32]. Reviewing the causality of an observed epidemiological association (correlation) requires a multidisciplinary approach based on the latest knowledge achieved across disciplines, and Hill’s viewpoints should only be used as a list for reference [35].

The method of evidence integration in this study differs from the previous methods that we referenced.

Firstly, there are differences in the purpose and final conclusion presenting from the results of evidence integration. The purpose of evidence integration in our study is to verify the *epidemiological correlation* between exposure and disease, which is the third requirement for presumption of causation in Article 5 of the Special Act. Scientific evaluation of causality can be expressed as a certain percentage or level of likelihood, reflecting the inherent uncertainty. However, a legal judgment, which determines the winner and the loser in a dispute between plaintiff and defendant, requires a binary decision about the presence or absence of *epidemiological correlation*. Meanwhile, the purpose of OHAT is to determine the harmfulness of a specific factor to support decisions on whether that factor needs to be controlled and to what degree. As such, evidence for different levels of control policies is assigned one of several categories. Whereas OHAT presents final conclusions about harmfulness as one of 4 levels, the final conclusions from our evidence integration method need to be presented as one of 2 levels: the presence or absence of an *epidemiological correlation* (3 levels if “not determined” is included).

Secondly, the assessment of risk of bias in individual epidemiological studies in the present study, is more lenient than existing evidence integration approaches for diagnostic or therapeutic methods. For example, ROBINS-I is a method of assessing the risk of bias in human clinical trials, cohorts or case-control studies observed during the usual treatment process [36]. These intervention studies are conducted in humans under the assumption that the intervention (or exposure) will confer potential benefit. Conversely, the exposure in our study is assumed to be a risk, rendering it impossible to conduct a trial with randomized or nonrandomized allocation of human participants. For this reason, most of the human studies that we review are non-intervention, non-trial, observational epidemiological studies. Observational studies are more susceptible to multiple types of bias compared to experimental studies, and thus if the same criteria were applied, the results from most human studies would be excluded from evidence integration. Therefore, it is necessary to apply more flexible assessment criteria to maximize the use of human observational studies in evidence synthesis [37].

Cases of severe health damages throughout history, such as the thalidomide tragedy, TCDD-related injuries in Vietnam, and the 9/11 attack on the Twin Towers, etc., demonstrate that even after exposure to the harmful, causative agent has stopped, various health damages can develop with different latencies even many decades later. These health damages may potentially affect the offspring of the victims, becoming a persistent social issue. The method for causal presumption developed in this study will need to be refined and supplemented based on new knowledge accumulated in the future, such as through long-term monitoring of the survivors of humidifier disinfectant exposure.

## CONCLUSION

The method of verifying *epidemiological correlation* established in this study not only includes all the viewpoints suggested in Hill’s method for reviewing causality, but also systematically accounts for the validity (quality) of all related studies.

In addition, by collectively considering all pieces of evidence, including epidemiology, toxicology, and clinical cases, this method not only reflects the advanced techniques and methodologies in each individual field, but also systematically reflects the scientific achievements and the integrated results of multiple disciplines.

The important viewpoints in Hill’s review of causality are all considered in the process of verifying *epidemiological correlation*. Whereas Hill’s method involves examining whether each viewpoint is satisfied, in the process of verifying *epidemiological correlation*, we consider the role, position and status of each viewpoint in determining the level of evidence. When assessing the level of confidence in the body of evidence, when profiling upgrading or downgrading factors affecting this level of confidence, and when determining the overall health effects (integrated level of evidence), the relevant viewpoints are repeatedly and multidimensionally reflected.

Furthermore, we account for research design factors that were not included in Hill’s viewpoints, such as the unit of the data (individuals vs. groups), whether a comparison group was included, imprecision considering sample size, residual confounding, and even publication bias.

Thus, this method of verifying *epidemiological correlation* (the weight-of-the-evidence approach) considers systematically, and in detail, diverse factors for reviewing causality, thereby reducing the risk of subjective judgments by the reviewer compared to previous methods using Hill’s viewpoints, which did not provide specific assessment standards for each viewpoint.

In this article, we present a systematic method for integrating a body of scientific evidence to verify *epidemiological correlation*, which is essential for presuming legal causation of health damages due to exposure to humidifier disinfectants. In the area of legal proof, this method does not exclude individual studies due to their limitations or uncertainty, but integrates them as part of a body of evidence to arrive at a conclusion that better reflects the truth of the evidence. This can be a model for future causal inference.

### Ethics statement

The study does not involve human or animal subjects but uses the existing literature.

## Figures and Tables

**Table 1.** Determination of epidemiological level of evidence of target health problem

Confidence level in body of evidence		Epidemiological level of evidence for target health problem^1^	
		Presence of health effects	Absence of health effects
High	→	Sufficient	Unrelated
Moderate	→	Suggestive	Unclassifiable
Low	→	Unclassifiable	
Very low	→		

1 Sufficient: Based on the epidemiological studies, there is a sufficiently high level of confidence that humidifier disinfectant exposure is associated with health problems, and thus the epidemiological level of evidence is high; Suggestive: Based on the epidemiological studies, there is a moderate level of confidence that humidifier disinfectant exposure is associated with health problems, and thus the epidemiological level of evidence is moderate; Unclassifiable: There is a shortage of epidemiological studies that can be used to determine whether humidifier disinfectant exposure is associated with health problems; Unrelated: Based on the epidemiological studies, there is a sufficiently high level of confidence that humidifier disinfectant exposure is not associated with health problems, and thus the epidemiological level of evidence is unrelated.

**Table 2.** Determination of toxicological evidence level for causality of health effect

Confidence level in body of evidence		Toxicological evidence level for causality of health effect^1^
High	→	Sufficient
Moderate	→	Suggestive
Unclassifiable	→	Unclassifiable
Low	→	Unrelated

1 Sufficient: There is a high level of overall evidence from exposure to adverse outcome, and thus the toxicological level of evidence for the causality between humidifier disinfectant exposure and health effects is sufficient; Suggestive: There is a moderate level of overall evidence from exposure to adverse outcome, and thus the toxicological level of evidence for the causality between humidifier disinfectant exposure and health effects is suggestive; Unclassifiable: There is a lack of toxicological studies that can be used to determine the confidence level of the overall evidence from exposure to adverse outcome, and thus the toxicological level of evidence for the causality between humidifier disinfectant exposure and health effects is unclassifiable; Unrelated: There is a low level of overall evidence from exposure to adverse outcome, and thus the toxicological level of evidence for the causality between humidifier disinfectant exposure and health effects is unrelated.

## SUPPLEMENTARY MATERIALS

Korean version is available at https://www.e-epih.org/.

Supplementary Material 1. ey content that needs to be summarized when reviewing selected epidemiological studies

Supplementary Material 2. Assessment of risk of bias in individual epidemiological studies

Supplementary Material 3. Determination of downgrading or upgrading factors in assessment of the confidence level of the cumulative evidence

Supplementary Material 4. Comparison of proof method in TCDD-related injury Cases between trial of appeal and trial at the Supreme Court

Supplementary Material 5. Comparison of Article 5 of the Special Act on Remedy for damage caused by humidifier disinfectants after the 2^nd^ amendment (March, 2020)

Supplementary Material 6. Types and evidence levels of epidemiological correlation

Supplementary Material 7. Process of weight of evidence assessment by using a body of evidence approach [1]

Supplementary Material 8. Comparison of the Hill’s viewpoints with the verification methods of the *Epidemiological Correlation*
