# Supplementary material for: Evidence integration on health damage for humidifier disinfectant exposure and legal presumption of causation
Source: Epidemiol Health. 2023 Oct 24;45:e2023095. doi: 10.4178/epih.e2023095 (PMC10876420; doi:10.4178/epih.e2023095)
Supplement: Supplementary file 1 [file epih-45-e2023095-Korean-Supplementary.pdf]

Open Access

Volume: 45, Article ID: e2023095
https://doi.org/10.4178/epih.e2023095

epiH Epidemiology and Health

SPECIAL ARTICLE

가습기살균제 건강피해의 근거종합과 법적 인과관계 추정

하미나[1]*, 박태현[2]*, 이종현[3], 김영희[4], 임정연[4], 백용욱[4], 유 솔[4], 정현미[4], 정규혁[5], 정해관[6],
가습기살균제 노출과 질환 간 역학적 상관관계 검토위원회

[1]단국대학교 의과대학 예방의학교실, [2]강원대학교 법학전문대학원, [3]EH R&C Co., [4]국립환경과학원 환경건강연구부 가습기살균제보건센터,
[5]성균관대학교 약학대학, [6]성균관대학교 의과대학

Evidence integration on health damage for humidifier disinfectant exposure and legal presumption of causation

Mina Ha[1]*, Taehyun Park[2]*, Jong-Hyun Lee[3], Younghee Kim[4], Jungyun Lim[4], Yong-Wook Baek[4], Sol Yu[4], Hyen-Mi Chung[4], Kyu Hyuck Chung[5], Hae-Kwan Cheong[6], Review Committee for the Epidemiological Correlations between Humidifier Disinfectants Exposure and Health Effects

[1]Department of Preventive Medicine, Dankook University College of Medicine, Cheonan, Korea; [2]Law School of Kangwon National University, Chuncheon, Korea; [3]EH R&C Co., Incheon, Korea; [4]Humidifier Disinfectant Health Center, Environmental Health Research Department, National Institute of Environmental Research, Incheon, Korea; [5]Sungkyunkwan University School of Pharmacy, Suwon, Korea; [6]Sungkyunkwan University School of Medicine, Suwon, Korea

OBJECTIVES: Inhalation exposure to humidifier disinfectants has resulted to various types of health damages in Korea. To determine the *epidemiological correlation* necessary for presuming the legal causation, we aimed to develop a method to synthesize the entire evidence.
METHODS: Epidemiological and toxicological studies are systematically reviewed. Target health problems are selected by criteria such as frequent complaints of claimants. Relevant epidemiologic studies are reviewed and the risk of bias and confidence level of the total evidence are evaluated. Toxicological literature reviews are conducted on three lines of evidence including hazard information, animal studies, and mechanistic studies, considering the source-to-exposure-to-outcome continuum. The confidence level of the body of evidence is then translated into the toxicological evidence levels for the causality between humidifier disinfectant exposure and health effects. Finally, the levels of epidemiological and toxicological evidence are synthesized.
RESULTS: Under the Special Act revised in 2020, if the history of exposure and the disease occurred/worsened after exposure were approved, and the *epidemiological correlation* between the exposure and disease was verified, the legal causation is presumed unless the company proves the evidence against it. The *epidemiological correlation* can be verified through epidemiological investigations, health monitoring, cohort investigations and/or toxicological studies. It is not simply as statistical association as understood in judicial precedents, but a general causation established by the evidence as a whole, i.e., through weight-of-the-evidence approach.
CONCLUSIONS: The weight-of-the-evidence approach differs from the conclusive single study approach and this systematic evidence integration can be used in presumption of causation.

KEY WORDS: Humidifier disinfectants, Presumption of causation, *Epidemiological correlation*, Weight-of-the-evidence approach, Systematic reviews, Evidence integration

Correspondence: Kyu Hyuck Chung
Sungkyunkwan University School of Pharmacy, 2066 Seobu-ro, Jangan-gu, Suwon 16419, Korea
E-mail: khchung@skku.edu

Co-correspondence: Hae-Kwan Cheong
Sungkyunkwan University School of Medicine, 2066 Seobu-ro, Jangan-gu, Suwon 16419, Korea
E-mail: hkcheong75@gmail.com

*Ha & Park contributed equally to this work as joint first authors.

Received: Feb 28, 2023 / Accepted: Sep 4, 2023 / Published: Oct 24, 2023

This article is available from: https://e-epih.org/

This is an open-access article distributed under the terms of the Creative Commons Attribution License (https://creativecommons.org/licenses/by/4.0/), which permits unrestricted use, distribution, and reproduction in any medium, provided the original work is properly cited.

© 2023, Korean Society of Epidemiology

서 론

가습기살균제는 가습기 수조 내 물때 발생과 미생물 번식 예방을 목적으로 1994년부터 2011년까지 국내에서 제조·판매되었다. 2006년부터 어린이에서 중증 호흡부전을 보이는 급성 간질성폐렴 유행이 보고되었고, 2008년에는 의학계 내에서 실태조사와 함께 보건당국에 바이러스 검사를 의뢰하였으나 원인을 밝히지 못하였다. 2011년 4월 한 대학병원에서 6명의 주산기 여성의 급성 중증폐질환을 보건당국에 보고하여 16명의 환자에 대한 역학조사를 실시하였고 그 결과 가습기살균제를 유력한 원인으로 지목하였다. 이후 동물시험에서 가습기살균제의 독성이 확인된 후 2011년 11월 보건당국은 가습기살균제 수거명령을 내리고, 이듬해 2월 가습기살균제가 집단적으로 발생한 폐손상의 원인임을 공식 발표하였다[1].

2013년 8월, 정부는 경제관계장관회의에서 위급한 어려움을 겪는 가습기살균제 피해자를 위한 경제적 지원을 결정하였다. 2014년 3월 가습기살균제 피해구제업무가 보건복지부에서 환경부로 이관되고 「환경보건법」에 기반하여 의료비와 장례비 지원이 시작되었다. 2016년 가습기살균제 제조·판매사에 대한 검찰수사와 국회 가습기살균제 국정조사특별위원회를 통한 진상규명 활동을 거쳐, 2017년 2월 「가습기살균제 피해구제를 위한 특별법」 (특별법)이 제정되었다. 특별법에 따라 가습기살균제의 원료와 제품의 제조·판매 사업자가 낸 분담금으로 특별구제계정이 조성되고 피해지원의 대상과 범위를 넓혔다[2].

2018년 8월 특별법의 1차 개정을 통해 특별구제계정 지원 대상자도 법상 피해자 지위를 부여하고, 특별구제계정 재원에 정부출연금을 추가하였다. 2020년 3월, 특별법 2차 개정을 통해 구제급여와 특별구제계정의 재원을 일원화하고, 건강피해를 '가습기살균제에 노출되어 발생하거나 악화된 생명 또는 건강상의 피해'로 포괄적으로 규정하여 피해의 범위를 확대하였다. 즉, 폐질환, 천식, 태아피해, 아동·성인 간질성폐질환, 기관지확장증, 폐렴 등의 특정 피해 질환을 앓는 경우가 아니더라도 폭넓은 구제가 가능하도록 하였다. 이에 따라 피해자의 가습기살균제 노출 전·후 건강상태를 종합적으로 검토하는 방식으로 건강피해 조사·판정체계의 전면 개편도 이루어졌다. 또한 가습기살균제에 노출된 사실, 노출 이후 질환의 발생·악화 사실, 노출과 질환 간 역학적 상관관계가 확인된 경우, 사업자의 반대사실의 증명이 없는 한 인과관계를 추정하도록 하여 손해배상소송에서 피해자의 입증 부담을 완화하였다[3]. 이는 기존의 특별법에서 가습기살균제 노출이 피해 질환을 유발하였다는 '상당한 개연성'의 입증이 소위 '비특이성 질환'에서는 쉽지 않았던 점이 보완된 것이다.

이에 따라 정부가 인정한 피해자의 수가 특별법 시행 전 280여명에 지나지 않았던 것이 특별법의 시행과 연 이은 두 차례 개정에 힘입어, 2023년 9월까지 정부에 피해구제를 신청한 사람 총 7,870명, 이 중 5,212명이 피해급여지급 결정을 받게 되었다. 가습기살균제 노출로 인해 질환의 발생이나 악화가 의심되는 사람은 언제든 피해신고를 할 수 있으며, 장기적 후유증도 피해대상이 되었다.

그러나 역학적 상관관계의 확인은 과학적 작업이 반드시 수반되어야 하는 일이어서 피해자 개개인이 입증하기 어렵다는 점을 감안하여, 환경부 장관이 직접 또는 전문 연구기관이 수행한 역학조사, 건강모니터링 그 밖의 코호트 조사와 독성연구 등을 통해 확인하도록 하였다[4].

이에 환경부 국립환경과학원은 학문 분야별 전문가들로 검토위원회를 구성하고 현존하는 다양한 과학적 근거들을 검토·평가하여 <역학적 상관관계 검토보고서>를 지속적으로 발간하는 의무를 지게 되었다[5]. 법적인 인과관계 추정요건의 하나인 역학적 상관관계를 확인하는 과학적 근거의 검토 과정은 투명하고 구체적으로 제시될 필요가 있다. 이를 위해 관련된 과학적 근거들을 체계적으로 검토, 평가하고 이 평가에 기반하여 특별법에 따른 '역학적 상관관계'를 확인하는 절차가 필요하다. 본 연구의 목적은 의학보건학 분야에서 체계적 문헌고찰 및 근거종합과 관련된 기존의 여러 방법론들을 참고하여 가습기살균제 노출과 건강영향 간 역학적 상관관계를 확인하기 위한 절차를 구체화하는 것이다. 본 논문에서 사용하는 '역학적 상관관계'는 특별법에서 정의하는 인과관계 추정요건인 법적 용어이다.

연구방법 및 결과

역학적 상관관계 검토보고서 발간 절차

가습기살균제 노출과 질환 간 역학적 상관관계 검토보고서 발간은 피해자의 소송을 지원하기 위하여 특별법에서 규정하고 있는 정부의 책무 중 하나이다[3]. 민·관으로 구성된 '가습기살균제 노출과 질환 간 역학적 상관관계 검토위원회' (검토위원회)가 관련 연구에 대한 체계적 문헌고찰과 근거 종합을 수행한다. 검토위원회는 환경노출학, 임상의학, 역학, 독성학 및 법학의 5개 학문분야별 전문위원회를 구성하고 전문위원회별로 전문가 3명 이상이 참여한다.

검토위원회는 이러한 검토과정을 거쳐, 특정 건강영향이 가습기살균제 노출과 관련성이 있는지를 평가하며, 최종적인 근거수준을 판단한다. 이 근거수준에 기반하여 법에서 정의하는 인과관계 추정을 위한 핵심요건으로서 '역학적 상관관계'가 확인되는지를 결정한다(Figure 1).

역학적 상관관계 확인을 위한 과학적 근거종합 체계

가습기살균제 노출에 의한 건강영향에 관한 역학적 상관관계의 확인은 전체증거 접근법에 따라 역학적 근거 및 동물실험과 기

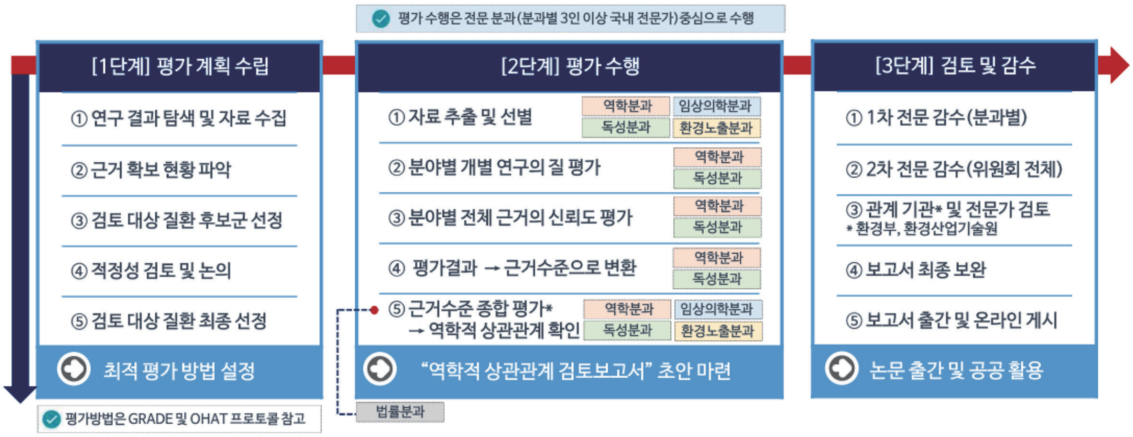

Figure 1. Publication process of the report on *epidemiological correlation* between humidifier disinfectants exposure and diseases. GRADE, Grading of Recommendations, Assessment, Development, and Evaluation; OHAT, Office of Health Assessment and Translation; MOE, Ministry of Environment; KEITI, Korea Environmental Industry & Technology Institute.

과학적 단계			법률적 단계
단계	역학적 근거 평가	독성학적 근거 평가	전체 증거 종합
1	대상 건강문제(질병) 선정	문헌고찰 개요작성 (PECO* 기술)	증거 수집 및 분류 (Assembling the evidence)
2	관련 연구 검색 및 결과 요약	관련 연구 선정 및 분류	
3	개별연구의 질평가<br>• 내적 타당도, 비뚤림위험 평가	근거계열의 독성학적 문헌고찰<br>• 노출 관련 유해정보<br>• 독성영향 관련 동물연구<br>• 작용기전 관련 기전연구	증거 비중 평가 (Weighing the evidence)
4	전체근거의 신뢰수준 평가<br>• 연구설계 예비평가<br>• 상·하향 요인 확인<br>• 신뢰수준 최종 평가	전체근거의 신뢰수준 평가<br>• AEP-AOP** 프레임워크에 의한 근거통합<br>• 신뢰성 및 관련성 평가	
5	역학적 근거수준 최종평가<br>• 건강문제의 근거수준으로 전환	독성학적 근거수준 최종평가<br>• 건강영향 인과성의 근거수준으로 전환	증거 종합 (Integrating the evidence)
6	역학적 상관관계 확인을 위한 역학적 근거수준 및 독성학적 근거수준의 종합		

Figure 2. Process to verify the *epidemiological correlation* between humidifier disinfectants exposure and health problems. *PECO; P (population): 대상집단, E (exposure): 노출요인, C (comparator): 비교집단, O (outcome): 독성영향. **AEP-AOP, 통합노출–독성발현경로(aggregate exposure pathway-adverse outcome pathway).

전연구 등 독성학적 근거를 모두 종합하여 판단한다. 역학적 상관관계 확인을 위한 절차는 대상 건강문제를 선정하는 첫 단계에서부터 역학적 근거와 독성학적 근거가 별개로 평가된 후 마지막에 종합되는 총 6단계로 진행된다(Figure 2).

근거종합 방법은 미국립독성프로그램(National Toxicological Program)에서 개발한 체계적 문헌고찰과 위해성평가의 근거종합에 관한 Office of Health Assessment and Translation (OHAT) 접근법 등을 기반으로[6], 가습기살균제의 노출과 건강영향 간의 역학적 상관관계를 확인하는 방법에 적합하도록 수정 변경하여 새로운 접근법을 확립하였다.

역학적 근거의 평가와 종합

가습기살균제 노출과 건강영향 간 역학적 연관성을 판단하기 위한 역학적 근거수준(level of evidence) 평가 절차는 ① 대상 건강문제 선정, ② 관련 연구의 검색 및 요약, ③ 개별 연구의 질(내적 타당도) 평가, ④ 전체근거(body of evidence)에 대한 신뢰수준(level of confidence) 예비평가, 상·하향 요인 확인 및 최종 신뢰수준 평가, ⑤ 역학적 근거수준 최종평가 등 5단계로 수행한다.

대상 건강문제의 선정

대상 건강문제는 (i) (피해신고자의 건강보험자료 분석 등을 통하여) 피해신고자가 가습기살균제 사용 이후에 다빈도로 수진한 질병, (ii) (전 국민 건강보험자료를 이용한 빅데이터 분석 등을 통하여) 우리나라 국민에서 가습기살균제 사용 기간에 사용하지 않은 기간에 비하여, 수진율, 발생률 또는 사망률이 높게 나타난 질병, (iii) 피해신고자가 다빈도로 호소하는 질병, (iv) 피해자나 노출확인자의 건강 추적(모니터링) 시 발견되는 (다빈도) 질병, (v) 그 외 역학적 상관관계에 대한 검토가 필요하다고 판단되는 질병인지를 고려하여 선정한다.

관련 연구의 검색과 결과 요약

관련된 검색 대상 연구로 환경부장관이 직접 수행한 연구 이외에, 국내·외에서 동료평가 학술지에 출판된 연구논문, 국제 또는 국내·외 정부기관, 규제기관, 자문기관, 연구기관 등에서 출판한 연구논문, 연구보고서, 규제기준, 지침 및 매뉴얼 등을 포함한다. 체계적 문헌고찰 방법에 따라 각종 전자 학술 데이터베이스에서 핵심어(keywords)를 중심으로 검색하여 얻어진 자료들의 제목과 초록을 검토하여 일차로 배제할 자료들을 걸러낸다. 중복된 자료, 노출 요인이 가습기살균제가 아닌 경우, 대상 건강영향에 대한 것이 아닌 경우, 원저(original study)가 아닌 경우 등은 배제한다. 일차 선택된 사람 대상 연구(역학 및 임상)에 관한 자료들은 원문을 검토하여 그 결과를 핵심적인 내용을 중심으로 정리한다(부록 1).

개별 연구의 질 평가

선정된 개별 연구의 요약이 끝나면 OHAT에서 제시하는 방법에 따라 비뚤림 위험(risk of bias)을 평가한다. 비뚤림은 체계적인 오류로서 연구 결과값을 실제의 값에서 더 멀어지게 하는 결과를 초래한다. 역학연구에서 발생할 수 있는 비뚤림에는 여러 가지 종류가 있으며, 개별 연구별로 이와 같은 비뚤림이 존재하는지를 평가하여, 연구의 내적타당도, 혹은 질을 평가한다. 가습기살균제 노출관련 역학연구에 무작위임상시험은 존재하지 않으므로, OHAT 방법에서 무작위임상시험에만 적용되는 항목(예, 무작위 배정, 눈가림법, 양측 눈가림법)은 제외한다[6].

각 비뚤림 항목에 대하여 비뚤림 위험이 거의 없음(++), 낮음(+), 있음(-), 높음(--)의 4가지로 평가하는데. 핵심요소인 측정비뚤림(노출평가 및 건강결과의 평가)과 교란비뚤림을 중심으로 평가하고, 다른 요소들을 함께 평가한다. 핵심요소 평가 결과가 ++/+ 이면서 다른 비뚤림 위험에서 대체로 ++/+ 이면 1등급, 핵심요소에서 -/-- 이면서 다른 비뚤림 위험에서 대체로 -/-- 인 경우 3등급, 나머지는 모두 2등급으로 판정한다(부록 2).

비뚤림 위험 평가는 우선 2인 이상의 역학자가 독립적으로 개별 연구에 대하여 평가한 후 역학 전문위원회와 외부위원이 포함된 5인 이상으로 구성된 역학 전문가회의에서 평가 결과를 검토·협의하여 최종평가를 내린다. 이때 3등급 연구는 이후의 근거종합 과정에서 배제한다.

역학연구의 전체근거에 대한 신뢰수준 평가

역학연구의 전체근거의 신뢰수준 평가는 연구설계를 중심으로 예비평가를 실시하고, 상향 혹은 하향요인을 확인한 결과를 반영하여 최종평가를 한다. 우선, 예비평가의 연구설계 항목은 (i) 노출이 실험연구 수준으로 통제되었는지, (ii) 노출이 건강 결과에 선행하는지, (iii) 건강 결과가 개인단위의 자료인지, (iv) 비교집단이 사용되었는지를 보아, '높음', '중간', '낮음', '매우 낮음' 등 4등급으로 구분한다. 따라서 통상 연구설계가 같으면 예비평가에서 동일한 등급을 받게 된다. 전체근거를 통합할 때 연구의 수가 많은 경우 메타분석을 할 수 있으나, 가습기살균제 역학연구의 경우 메타분석은 어렵다. 왜냐하면, 가습기살균제 노출의 정의가, 가습기살균제 수거조치 전·후의 시기(전 국민 코호트), 가습기살균제 사용 시작 시점과 종료 시점(피해신고자 코호트) 혹은 가습기살균제 노출량에 관한 간접적 지표(총 사용 기간, 하루 평균 사용 시간 등)나 개인별 노출량을 직접 추정하여 산출한 지표 등으로 연구별로 다르기 때문이다.

다음으로 비뚤림 위험, 설명되지 않는 비일관성, 간접성, 비정밀성, 출판비뚤림과 관련된 하향요인과, 효과의 크기, 양-반응 관계, 잔여교란효과, 인구집단 간 일관성과 관련된 상향요인이 있는지를 검토하여 등급을 올리거나 낮춘다[6,7] (부록 3). 등급은 예비평가와 동일한 4가지로, 노출과 건강결과 간 관련성이 실제인지에 대하여 높음(4+, 실제일 가능성이 높음), 중간(3+, 실제일 가능성이 있음), 낮음(2+, 실제와 다를 가능성이 있음), 매우 낮음(1+ 이하, 실제와 다를 가능성이 높음)으로 구분한다.

여러 등급의 최종평가 결과가 있다면, 건강영향의 존재 여부와 무관하게 가장 높게 평가된 신뢰수준을 역학연구의 전체근거에 대한 신뢰수준으로 채택한다.

대상 건강문제의 역학적 근거수준 판단

전체근거에서 역학연구의 결과가 건강영향이 있는 경우와 건강영향이 없는 경우로 구분하여 역학연구의 전체근거 신뢰수준을

Table 1. Determination of epidemiological level of evidence of target health problem

전체근거 신뢰수준		건강문제의 역학적 근거수준[1]	
		건강영향이 있는 경우	건강영향이 없는 경우
높음	→	충분	관련성 없음
중간	→	시사적	유보
낮음	→	유보	
매우 낮음	→		

[1]충분(sufficient): 가습기살균제 노출과 건강문제 간 연관성에 대한 높은 신뢰수준의 역학연구가 충분하므로 역학적 근거수준이 높음; 시사적(suggestive): 가습기살균제 노출과 건강문제 간 연관성에 대한 보통 신뢰수준의 역학연구가 있으므로 역학적 근거수준이 보통임; 유보(unclassified): 가습기살균제 노출과 건강문제 간 연관성을 판단하는 데 활용할 수 있는 역학연구가 충분하지 않음; 관련성 없음(unrelated): 가습기살균제 노출과 건강문제 간 연관성이 없음에 대한 높은 신뢰수준의 역학연구가 충분하므로 역학적 근거수준이 높음.

대상 건강문제에 대한 역학적 근거수준으로 전환한다. 최종 건강영향 유무에 따른 역학적 근거수준은 충분, 시사적, 유보, 관련성 없음의 4단계로 구분된다. 건강영향이 있는 경우, 전체근거의 신뢰수준이 '높음'이면 역학적 근거수준은 '충분', '중간'이면 '시사적', '낮음'이나 '매우 낮음'이면 '유보'로 분류된다. 건강영향이 없는 경우에는 전체근거의 신뢰수준이 '높음'인 경우에만 역학적 근거수준이 '관련성 없음'으로 분류되며, 나머지는 모두 '유보'로 분류된다(Table 1).

독성학적 근거의 평가와 종합

화학물질 노출과 질병 간 인과관계에 대한 결론을 확고하게 하기 위해서는 독성학과 역학의 공동작업이 필수적이다[8]. 이때 독성학의 중요한 역할은 생물학적 개연성(biological plausibility)의 증명이다. 이는 잠재적 원인과 질병 발생 간 연관성에 대한 연구결과가 기존에 알려진 생물학적 지식, 즉 독성학의 원리와 일치하는지를 판단할 수 있는 근거를 제시한다[9].

기존에는 동물연구(animal studies)에 주로 의존하였으나 인체 또는 동물 유래의 조직이나 세포 등을 이용하는 비동물 연구(non-animal studies)가 발전하면서 다양한 독성 데이터의 활용이 가능하게 되었다. 특히 최근에는 발생원-노출-용량-독성발현의 인과적 연속성을 평가하는 통합노출경로(aggregate exposure pathway, AEP)와 독성발현경로(adverse outcome pathway, AOP)의 개념이 도입되어 유해성 판단에 대한 인체영향과의 관련성을 높이고 있다[10].

가습기살균제와 건강영향 간 인과성에 대한 독성학적 근거수준의 평가 절차는 (1) 문헌고찰의 개요 작성, (2) 관련 연구의 선정 및 분류, (3) 근거계열에 대한 독성학적 문헌고찰, (4) 전체근거의 신뢰수준 평가, (5) 독성학적 근거수준 최종 평가의 단계로 수행한다.

문헌고찰의 개요 작성

대상 건강영향에 대한 독성학적 근거수준을 평가하기 위하여, 독성학적 문헌의 체계적 고찰 범위를 정의하고 PECO (Population 대상집단-Exposure 노출요인-Comparator 비교집단-Outcome 독성영향)를 기술한다. 이는 문헌고찰의 목표를 명확하게 정의하고 범위를 한정하여 체계적으로 조사함으로써 결과 해석의 타당성과 신뢰성을 향상시킨다.

관련 연구의 선정 및 분류

각종 전자 학술 데이터베이스에서 핵심어(keywords)로 가습기살균제와 성분, 건강영향 관련 질병, 독성영향과 기전 등을 중심으로 검색한다. 선정한 문헌은 ① 가습기살균제 성분의 물리·화학적 특성, 일반독성 및 노출 관련 '유해정보', ② 실험동물을 사용하여 독성학적 연구를 수행한 '동물연구', ③ 세포 또는 조직 등을 사용하여 작용모드를 연구한 '기전연구' 등 세 가지 근거계열(line of evidence)로 분류한다. 연구논문(research article)과 종설논문(review article) 그리고 관련 보고서 및 발표자료 등에서 PECO를 고려하여 관련 데이터를 추출한다.

근거계열(line of evidence)에 대한 독성학적 문헌고찰

노출 관련 유해정보의 체계적 문헌고찰

유해정보는 Organization for Economic, Cooperation and Development (OECD), European Chemical Agency, Environmental Protection Agency를 포함한 국내·외 규제기관에서 제공하는 물리·화학적 특성, 안정성 및 반응성, 일반 독성, 특히 흡입독성(단회 및 반복 노출시험) 등에 관한 정보를 수집한다. 또한 실제 가습기살균제 사용 실태를 고려한 시험조건에서 수행된 연구결과와 호흡기 침착량 예측 및 노출수준 등에 관한 연구결과로부터 데이터를 추출한다.

통합노출경로(AEP) 프레임워크[11]를 활용하여 유해정보의 체계적 문헌고찰을 수행하고 노출 관련 근거를 분석한다. 이를 위해 추출한 노출 관련 모든 데이터 정보를 활용하여, 발생원에서 공기 중에 방출된 가습기살균제 성분이 체내에 유입되어 표적노출부위에 노출되기까지의 경로를 작성한다.

독성영향 관련 동물연구의 체계적 문헌고찰

동물연구의 체계적 문헌고찰을 위해 랫드 또는 마우스 등 실험동물을 사용하여 인체 건강영향을 간접적으로 평가한 문헌을 수집하고, 일반독성(체중 변화 등), 조직병리학적 또는 영상의학적 소견, 생체지표 변화 등 독성영향에 관한 데이터를 추출한다.

독성발현경로(AOP) 프레임워크를 활용하여 동물연구의 체계적 고찰을 수행하고 독성영향 관련 근거를 분석한다. 이를 위해 시험물질, 실험동물, 연구설계, 시험수행 및 통계분석, 시험결과 및

결론 등 개별연구의 신뢰성[12]과 대상 건강영향과의 관련성을 확인한다.

작용모드 관련 기전연구의 체계적 문헌고찰

기전연구의 체계적 고찰을 위해 인체 또는 동물에서 유래한 세포 또는 조직을 이용하여 작용모드를 연구한 문헌을 수집하고, 분자개시사건(molecular initiation event), 핵심사건(key events), 독성발현(adverse outcome) 등 작용모드와 관련한 데이터를 추출한다.

독성발현경로(AOP) 프레임워크[13]를 활용하여 기전연구의 체계적 문헌고찰을 수행하고 작용모드 관련 근거를 분석한다. 이를 위해 추출한 데이터를 활용하여 가습기살균제 성분이 노출표적 부위에서 거대분자와 반응이 시작되고, 세포소기관, 세포, 조직 및 기관의 단계별 생체반응을 거쳐 독성이 발현되는 경로를 작성한다.

전체근거의 신뢰수준 평가

가습기살균제가 건강영향과 관련된 독성발현의 원인인지를 판단하기 위해 세 가지 근거계열(유해정보, 동물연구, 기전연구)을 AEP-AOP 프레임워크를 활용하여 발생-노출-독성발현에 대한 전체근거(body of evidence)로 통합한다. 전체근거의 신뢰수준은 신뢰성(reliability) 및 관련성(relevance)을 기준으로 평가한다. 신뢰성은 브래드포드 힐(Bradford Hill)의 기준(관점)을 참고하며, AEP-AOP 네트워크의 핵심사건(key event)과 독성발현(Adverse outcome)을 강도(strength) 및 양-반응관계(dose-response) 등의 측면에서 연구들 간의 일관성(consistency)에 대해 평가한다. 관련성은 OECD의 AOP 평가지침[14]을 참고하며, 가설로 설정된 대상 건강영향의 발생기전과 비교하여 경로의 적절성(adequacy)을 평가하고, 핵심사건의 필수성(essentiality)과 데이터의 충분성(sufficiency) 등이 이를 지지하는지를 평가한다. 신뢰수준의 평가는 검토위원회의 독성분과 전문위원회가 수행하며 '높음', '중간', '낮음', 그리고 연구가 부족하거나 진행 중인 경우 '유보'로 구분한다.

독성학적 근거수준 판단

세 가지 근거계열을 통합하여 평가한 전체근거의 신뢰수준을 독성학적 근거수준으로 전환하여 가습기살균제 노출과 건강영향 간 인과성을 판단한다. 이는 가습기살균제에 관한 유해정보, 동물연구, 기전연구의 전체근거를 통합적으로 평가함으로써 실험적 증거(experimental evidence)에 대한 생물학적 타당성을 향상하고, 인과적 증거(causal evidence)에 대한 인체 관련성을 강화한다. 건강영향 인과성의 독성학적 근거수준은 충분, 시사적, 유보, 관련성 없음의 4단계로 구분한다. 전체근거의 신뢰수준이 '높음'이면 독성학적 근거수준은 '충분', '중간'이면 '시사적', '낮음'이면 '관련성 없음', '유보'이면 '유보'로 분류된다(Table 2).

Table 2. Determination of toxicological evidence level for causality of health effect

전체근거 신뢰수준		건강영향 인과성의 독성학적 근거수준[1]
높음	→	충분
중간	→	시사적
유보	→	유보
낮음	→	관련성 없음

[1]충분(sufficient): 가습기살균제의 노출에서 독성발현까지 전체근거의 신뢰수준이 높으므로, 가습기살균제 노출과 건강영향 간 인과성에 대한 독성학적 근거수준이 충분함; 시사적(suggestive): 가습기살균제의 노출에서 독성발현까지 전체근거의 신뢰수준이 보통이므로, 가습기살균제 노출과 건강영향 간 인과성에 대한 독성학적 근거수준이 시사적임; 유보(unclassified): 가습기살균제의 노출에서 독성발현까지 전체근거의 신뢰수준을 판단하는데 활용할 수 있는 독성학적 연구가 부족하므로, 독성학적 근거수준을 분류할 수 없음; 관련성 없음(unrelated): 가습기살균제의 노출에서 독성발현까지 전체근거의 신뢰수준이 낮으므로, 가습기살균제 노출과 건강영향 간 인과성에 대한 독성학적 근거수준이 관련성 없음.

전체 과학적 근거의 종합

특별법 제5조에 따른 역학적 상관관계는 법적 인과관계에서 '일반적 인과관계'로 해석하며, 가습기살균제 노출의 건강영향에 관한 역학적 근거수준과 독성학적 근거수준을 종합하여 판단한다.

역학적 상관관계는 '역학적 상관관계 있음이 확인', '역학적 상관관계 없음이 확인' 또는 '역학적 상관관계 확인이 유보'되는 총 3가지 경우로 구분된다. 근거종합을 통해 '역학적 상관관계가 있음이 확인'되는 경우는 ① 역학적 근거수준이 충분한 경우 또는 ② 역학적 근거수준이 시사적이나 독성학적 근거가 충분/시사적인 경우이다. '역학적 상관관계가 없음이 확인'되는 경우는 역학적 근거 및 독성학적 근거 모두 관련성 없음인 경우가 해당된다.

그 외 경우는 모두 역학적 상관관계 확인이 유보된다. 다만 독성학적 근거는 충분하나, 피해 유형이 드물거나 질병 발생률이 낮아 관찰적 역학연구가 수행되기 어려운 경우, 가습기살균제 노출과 관련된 임상 사례가 존재한다면, 근거종합의 등급이 상향 조정될 수 있다(Figure 3).

특별법에서 과학적 근거종합에 의한 법적 인과관계 증명

역학연구를 통한 법적 인과관계의 증명

대법원 판례에 따르면, 불법행위의 성립요건인 인과관계는 반드시 의학적·자연과학적으로 명백히 증명되어야 하는 것은 아니고, 경험칙과 사회통념에 따라 합리적인 추론을 통하여 인정될 수 있다. 법원은 공해소송에서 인과관계의 증명 정도를 '확실성'에서 상당 정도의 가능성을 의미하는 '개연성'으로 완화하였다. 개연성 법리에 따르면, 가해자가 어떠한 유해한 원인물질을 배출하고 그것이 피해물건에 도달하여 손해가 발생하였다면 가해자가 그것이

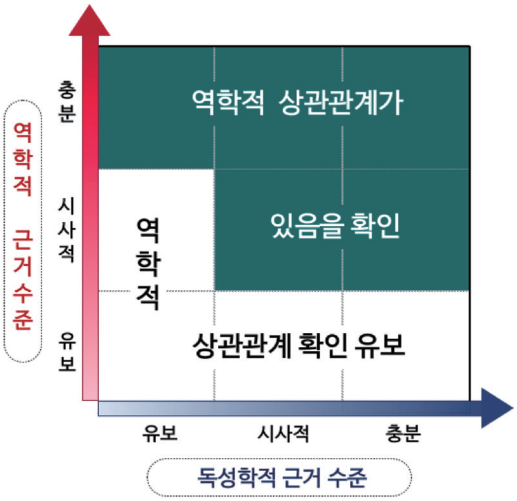

Figure 3. Method to verify the *epidemiological correlation* between humidifier disinfectants exposure and health damages based on the integrated evidence levels of epidemiology and toxicology.

무해함을 입증하지 못하는 한 책임을 면할 수 없다[15]. 생명, 신체 등 건강상 손해배상소송에서는 2단계 인과관계의 증명이 요구된다. 일반적으로 인체가 당해 유해물질에 노출될 경우 문제된 질병이 야기될 수 있다는 일반적 인과관계와, 당해 피해자가 당해 유해물질에 노출된 후 질병이 발생하였다는 개별적 인과관계가 그것이다[16].

역학은 인구집단의 건강과 질병에 관한 연구로 일반적 인과관계의 규명에 중점을 두고 있어, 역학연구에서 제시된 증거는 개별 원고의 인과성을 판단하는 것과 단순히 동일하지 않다. 역학연구를 통해 연관성이 확인되고, 비뚤림 등 연구의 타당성을 훼손할 수 있는 요인이 개입되지 않았음이 인정되면, 연관성이 원인-효과 관계인지를 추론하게 된다. 이러한 인과성 추론에는 통상 브래드포드 힐(Bradford Hill)이 제시한 9가지 관점을 적용하는데, 제시된 관점들 중 시간적 선후관계만이 필수적이며 나머지는 해당 관점을 충족하여도 인과적 관계가 아니거나 인과적 관계임에도 해당 관점을 충족하지 못하는 경우가 있다. 그러나 충족되는 관점이 많을수록 역학적 연관성이 인과관계일 가능성이 높아진다[17].

그러나 미국 법원은 기본적으로 개별 원고의 피해에 대한 인과관계를 판단해야 하므로, 역학적 증거가 개별적 인과관계의 증거로 활용되려면, 증거로서 충분히 엄격하여야 할 뿐 아니라(허용성), 개인에서의 개연성을 인정할 수 있을 정도의 충분한 증명력(충분성)을 갖추어야 하는 것으로 보고 있다[18]. 한편 한국 법원은 개인에서 인과성을 판단할 때, 인구집단을 대상으로 한 통계로부터 개인에 관한 통계적 추론을 하는 것은 부적절하거나, 원인과 결과가 일대일로 대응하지 않는 비특이성 질환에서는 역학적 인과관계를 곧바로 개인에게 적용하기에 충분하지 않다고 본다[15,16,19,20].

고엽제 사건의 고등법원에서는 역학적으로 인과관계가 확인되었다면, 개개 피해자에서 당해 유해물질이 도달한 후 당해 질병이 발생한 사실로부터 개별적 인과관계에서도 상당한 개연성이 있다고 인정할 수 있다고 하였다[21]. 이와는 달리 대법원에서는 일반적 인과관계는 개인에서 그 위험인자로 인하여 해당 질병이 발생하였을 가능성이 얼마나 되는지를 추론할 수 있을 뿐이라고 하였다[16]. 이에 따라 특이성 질환과 비특이성 질환을 구분하여, 비특이성 질환에서는 노출된 집단이 질환에 걸린 비율이 노출되지 않은 집단에 비하여 상당히 초과한다는 점을 증명할 뿐 아니라, 그 집단에 속한 개인(원고)의 특징과 상태(노출시기와 노출정도, 발병시기, 노출 전 건강상태, 생활습관, 가족력, 질병상태의 변화 등)를 살펴보아, 그 위험인자로 인해 그 비특이성 질환이 유발되었을 개연성을 추가로 증명하여야 한다고 하였다(부록 4).

특별법에서 역학적 상관관계의 확인

특별법 제5조의 인과관계의 추정은, 생명 또는 건강상의 피해가 가습기살균제에 의한 것으로 볼만한 '상당한 개연성이 있는 때'로부터, 2020년 3월 개정을 통해 '가습기살균제 노출 이후 발생하였거나 악화된 질환이 가습기살균제 노출과 역학적 상관관계가 있음이 확인된 경우'로 변경되어, 피해자의 입증 부담이 완화되었다[3]. 다만, 사업자가 '다른 원인으로 인하여 그 피해가 발생하였다'라는 사실을 증명하면 이 추정은 깨진다(부록 5).

즉, 특별법에서는 가습기살균제 노출이 해당 질환을 일으키거나 악화할 수 있는 일반적 가능성(위험)이 확인되면 개별적 인과관계를 따지지 아니하고 원고가 가습기살균제에 노출된 사실과 노출 이후 질환이 발생하였거나 악화되었다는 사실을 증명하는 것으로(사업자의 반대사실의 증명이 없다면) 인과관계 추정이 완료된다(Figure 4).

이러한 법 개정은 피해자의 입장에서 관련정보 및 전문적인 지식의 부족 등으로 상당한 개연성을 입증하는 것이 쉽지 않아 인과관계의 추정요건을 완화하기 위한 것이다. 애초 발의된 다수의 개정안에서는 가습기살균제에 노출된 사실과 노출 이후 질환이 발생하였거나 기존질환이 악화된 사실만으로 인과관계가 추정되는 것으로 하였는데, 국회 환경노동위원회 대안에서 사업자가 피해자의 건강피해가 다른 원인으로 인한 것이라는 증명을 한다면 그러하지 아니하다는 조건이 추가되었다[22]. 법제사법위원회에서는 법무부와 법원행정처 등이 인과관계의 추정범위가 지나치게 넓혀져 자기책임의 원리에 반할 우려가 있음을 제기하였고, 이를 반영하여 가습기살균제 노출과 질환 간 역학적 상관관계가 있음이 확인되어야 한다는 조건이 추가된 것이다[23].

전체증거 접근법에 의한 법적 인과관계의 증명

특별법 시행령 제2조에서는 역학적 상관관계의 확인을 위해 역학조사는 물론 건강모니터링, 독성연구 등(환경부장관이 직접 또

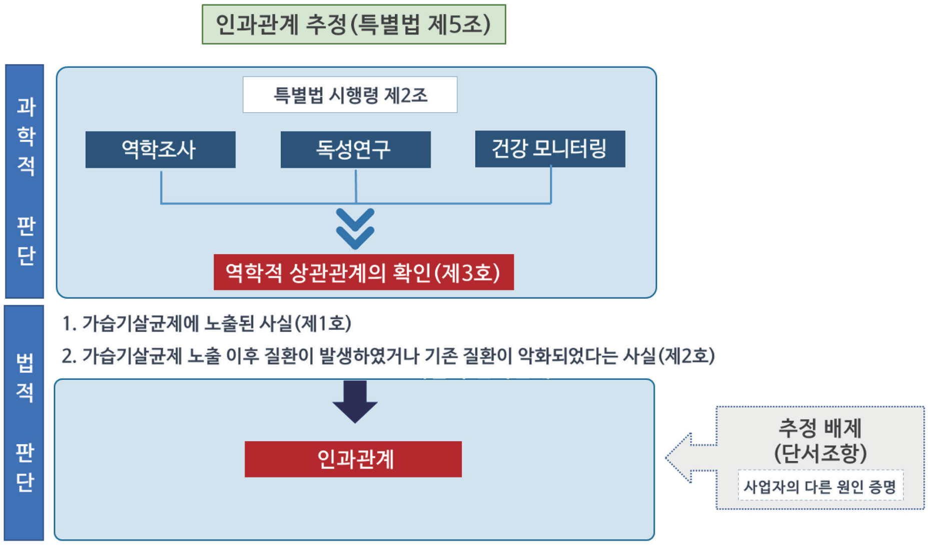

Figure 4. Process of presumption of causation under the Special Act on Remedy for Damage Caused by Humidifier Disinfectants, Article 5.

는 전문연구기관이 수행한) 이용 가능한 다양한 증거방법에 기반하도록 하고 있다[4]. 이러한 특별법령은 두 가지 함의를 제시한다. 첫째, 특별법상 역학적 상관관계란 종래 판례에서 말한 단순한 통계적 연관성으로만 이해해서는 안 된다는 것이다. 둘째, 특별법의 역학적 상관관계는(법령에 따라 이용 가능한) 다양한 증거방법에 대한 증거력 평가 후 종합 검토를 거쳐 확인해야 한다는 것이다.

법원은 역학적 상관관계를 인구집단에서 확인된 통계적 연관성으로 이해하고, 높은 강도의 통계적 연관성으로 일반적 인과관계를 인정하고 있다[16]. 그러나 과학적으로 역학적 상관관계의 개념은 통계적 연관성에 국한되지 않고, 상관성(correlation), 연관성(association), 인과성(causation)을 모두 포함하는 넓은 개념이며, 상관성→연관성→인과성 순으로 역학적 상관관계에 대한 근거의 수준이 높아진다(부록 6). 이러한 점에서 특별법의 역학적 상관관계의 개념은 가습기살균제 노출과 질환 간 관계(relationship)에서 단순히 관련성 지표에서 높은 수치를 나타내는 경우(강한 통계적 관련성)만을 포함하는 것이 아니라, 다양한 유형의 관계와 그 근거수준의 폭넓은 범위를 모두 고려한다. 즉, 해당 피해질환의 발생 혹은 악화에 미친 가습기살균제 노출의 다양한 역할, 예를 들어 주요한 직접적 원인만이 아니라, 간접적, 보조적, 주변환경적 원인으로, 또는 다른 원인과의 상호작용을 통한 기여 등을 모두 포함하는 것으로 이해되어야 한다.

화학물질 등 환경유해인자 노출에 따른 집단 피해가 발생한 사건에서는 먼저 역학조사를 통하여 집단 차원에서 인체영향과 위험인자와의 관련성을 확인하는 것이 필요하다. 인과성 확인에서 황금기준으로 인정되는 무작위임상시험(randomized clinical trial)과 같은 실험적 역학조사는 가습기살균제와 같은 유해인자에 대해서는 실시할 수 없다. 뿐만 아니라 질병이 희소하거나 관찰 대상자의 수가 적어 통계적 검정력을 확보할 수 없는 경우나 적절한 연구 집단이나 자료원이 존재하지 않는 경우에는 관찰적 역학조사도 수행하기 어렵다. 이때는 해당 피해사례나 사례군의 노출 특성 및 임상적 경과와 양상, 건강모니터링과 같은 임상적 추적 관찰 자료와 동물실험을 포함한 생체 내 및 생체 외 실험의 독성학적 연구 등, 가용한 모든 근거들을 종합적으로 고찰함으로써 특정 위험요인에 의한 건강영향 발생 가능성 등을 확인하여야 한다.

다른 한편 법원은 법에서 인과관계를 '사실적(또는 자연적)' 인과관계와 '법률적(또는 이념적)' 인과관계(즉 상당인과관계)로 구분하여 이해하며, 법률적 인과관계는 사실적 인과관계를 전제하고 있다[24]. 다만, "사실적 인과관계 유무 검토는 위 법률적 인과관계 유무의 판단에 필요 충분한 정도에 그쳐야 할 것"이므로, 법(재판) 영역에서 사실적 인과관계의 증명 정도는 '규범적 관점'에서 정해지게 된다[25]. 사실적 인과관계에 대한 판단에서는 자연과학적 또는 의학적 증거에 의한 증명이 필요하다. 자연과학적 또는 (임상)의학적 증거들로 사실적 인과관계를 추론하는 두가지 방법으로 단일증거 접근법과 전체증거 접근법이 있다. 단일증거 접근법은 인과관계를 완전히 지지하는 결정적인 단일연구(a single dispositive study)를 찾으려는 접근법으로 주로 판사가 취하고 있는 태도이다[26]. 이 접근법은 다수의 연구에서 나온 근거들을 종합하여 보지 않고 각 연구를 별개로 검토하여 개별연구가 가진 제한점이나 불확실성을 이유로 증거로서 가치를 인정하지 않는다[27]. 이와 달리 전체증거 접근법은 과학적 증거(지식)는 본질상 누적적임을 인정하는 전제에서 어떤 결론을 단정하고 있지는 않지만 이를 시사하는 다수의 연구 결과에서 과학적 간접사실(suggestions)을 추론하려는 접근법으로 과학자나 환경이나 보건 문제를 관할하는 전문 행정청이 취하는 태도이다[28].

따라서 특별법에서 사실적(자연적) 인과관계 판단의 주요 부분인 역학적 상관관계는 전체증거 접근법에 따라 개별 증거의 가치, 나머지 증거와 부합성 여부 및 그것이 전체증거에서 차지하는 비중 등의 평가를 통해 확인되어야 한다는 것을 의미한다[29] (부록 7). 특별법상 역학적 상관관계는 가용한 다양한 증거에 대한 종합적 검토·평가를 통해 확인한 것으로 판례에서 말하는 통계적 연관성을 포함하되 이를 넘어서는 개념이다. 즉 역학조사 결과(findings)의 증거력은 건강모니터링이나 동물실험, 독성연구 그 밖의 다른 조사·연구에 의해 증강 또는 보완될 수 있다. 이러한 증강 또는 보완이라는 전체증거의 통합적 검토 과정(=전체증거의 종합 과정)을 거쳐 도출, 해석하고 평가한 결과가 곧 역학적 상관관계의 확인과정이다.

고 찰

본 연구에서 법적 인과관계 추정 요건인 역학적 상관관계를 확인하는 과정을 체계적 문헌고찰과 역학, 독성학, 임상의학, 환경노출학, 법학 등의 다학제적인 근거를 종합하는 방법으로 구체화하였다. 특히 미국립독성프로그램에서 개발한 체계적 문헌고찰과 위해성평가의 근거종합에 관한 OHAT 접근법을 응용하여 역학과 독성학의 과학적 근거를 종합하는 새로운 접근법을 제시하였다 (Figure 5).

본 연구에서 제시한 역학적 상관관계를 확인하는 과정은 과거 Hill이 제시한 관점을 활용하되(부록 8), 여러가지 면에서 차별성을 가진다.

관찰된 역학적 관련성이 인과적인지를 판단하는 관점이 Hill (1965년)에 의해 제시되었다[30]. 그러나 그간 의학 및 생물학은 눈부시게 발전하였고 질병 원인에 대한 이해의 수준은 당시에 비하여 훨씬 높다. Hill의 검토 관점도 이러한 최신 의학과 생물학의 발전을 반영하여 지속적으로 수정되어, 최근에는 9가지(시간적 선후관계, 연관성의 강도, 용량-반응 관계, 결과의 재현성, 생물학적 개연성, 다른 해석의 가능성 고려, 노출의 종료, 기존 지식과 일관성, 연관성의 특이성)로 제시되고 있다[31].

무엇보다 중요한 것은, 이들 관점을 절대적인 기준으로 판단하지 않아야 한다는 것이다. 다수의 자료원에서 그 인과성을 지지하는 여러 형태의 증거가 있을 경우, 인과성에 대한 결론은 크게 강화될 수 있다. 그러나 이 모든 관점에 정확히 들어맞는 경우는 많지 않으며, 실제로는 한두 가지 관점에서 충족되고 대략적인 증거 패턴으로 판단되는 경우가 더 많다. 시간적 선후관계만이 인과성 판단에 필수적인 관점이며 나머지는 참고할 사항이다[32].

우선, 양-반응 관계에 있어서는 일정 수준 이하의 노출에서는 반응이 일어나지 않는 역치(threshold)가 있거나, 노출량 증가에 따른 반응의 정도가 U자형, S자형 혹은 J자형과 같은 비선형적인 관계를 보이는 경우가 드물지 않다. 이때는, 원인-결과 사이의 관련성이 인과적임에도 일부 혹은 전체 노출 범위에서 선형적인 양-반응 관계가 나타나지 않을 수 있다. 뿐만 아니라, 연령이나 성별 등에 따라 감수성에 차이가 있거나, 다른 요인들과 상승(synergistic) 혹은 길항(antagonistic) 작용을 하는 경우 양-반응 관계는 전혀 다른 패턴을 보일 수 있다[32]. 이러한 측면을 고려하지 않았을 때 인과적 관련성에 대해 잘못된 판단이 내려질 수 있다.

역학적 관찰 결과가 때로 최신의 생물학적 지식을 앞서갈 수 있어 인과적 관계임에도 이를 생물학적으로 설명하기 어려운 경우도 있다. 역학의 역사에서 고전으로 일컬어지는 1800년대 John Snow의 런던 콜레라 역학조사가 이를 분명하게 보여주고 있다. Hill 스스로도 이 생물학적 개연성의 관점은 현재의 지식수준(current state of knowledge) 자체가 한계점이라고 인정하고 있다[17].

다른 해석의 가능성 고려는 흔히 잘못 적용되는 관점이다. 이 관점은 제3의 요인의 역할을 교란효과에 국한하여 이해할 뿐 아니라 병원체에서 출발한 단일 병인론이 근저에 있는 것으로 보인다[30]. 하나의 질병에는 하나의 원인이 있다고 간주하는 이른바 단일병인론은 현미경의 개발, 박테리아의 발견과 비약적인 생의학의 발전이 있었던 근대 르네상스 시대의 개념이다. 만성질환이 주를 이루는 현대의 병인론은 어떤 질병도 하나의 원인에 의해 발생한다고 보지 않으며, 이것은 병원체로 인한 감염병에서도 그러하다[31].

건강과 질병 상태는 연령, 성, 유전적 소인뿐 아니라 영양·면역 상태 등의 개인적 조건, 물리·화학적 환경과 사회 제도적 환경을 둘러싼 다층적이고 다양한 요인이 함께 복잡하게 작용하여 결정된다[31,32]. 각 요인들은 노출 후 질병이 발생하는 경로의 다양한 시점에서 다양한 역할로 질병 발생에 기여한다. 제3의 요인은 원인-결과 사이에서 단지 교란효과만을 일으키는 것은 아니다. 제3의 요인은 관심 요인(주요 노출 요인)과 함께 상호작용하여 상가(additive)·상승(synergistic)·보완(complementary) 효과 등을 유발할 수도 있고 이때는 제3의 요인이 존재함으로써 관심 요인이 질병 발생에 기여할 수 있게 된다. 따라서 다른 해석의 가능성을 고려해야 한다는 Hill의 관점을 마치 관심 요인을 제외한 다른 요인의 역할이 모두 배제되어야 하는 것으로 해석한다면 실제의 인과관계가 부정되는 결과가 초래될 것이다. 즉, 관심 요인 이외의 다른 유력한 요인이 존재할 경우, 대립적·선택적으로 원인 요인을 판단할 것이 아니라, 질병 발생의 전 과정에서 각 요인들의 역할, 상호작용과 기여 정도를 종합적으로 고려하여야 함을 의미한다.

'노출의 중단'은 관심 요인의 노출이 중단되는 경우 질병이 현저히 줄어들거나 더 이상 발생하지 않는다면 그 인과관계가 강력히 지지될 수 있다는 것이다. 노출 중단의 효과는 실험을 통해 관찰할 수 있지만, 자연 혹은 사회 환경적으로 발생한 어떤 사건이 마치 실험적으로 노출을 중단시킨 것 같은 상황이 되고 이를 관찰할 수 있다. 대표적인 예가 2011년 11월 가습기살균제의 수거 조치 이

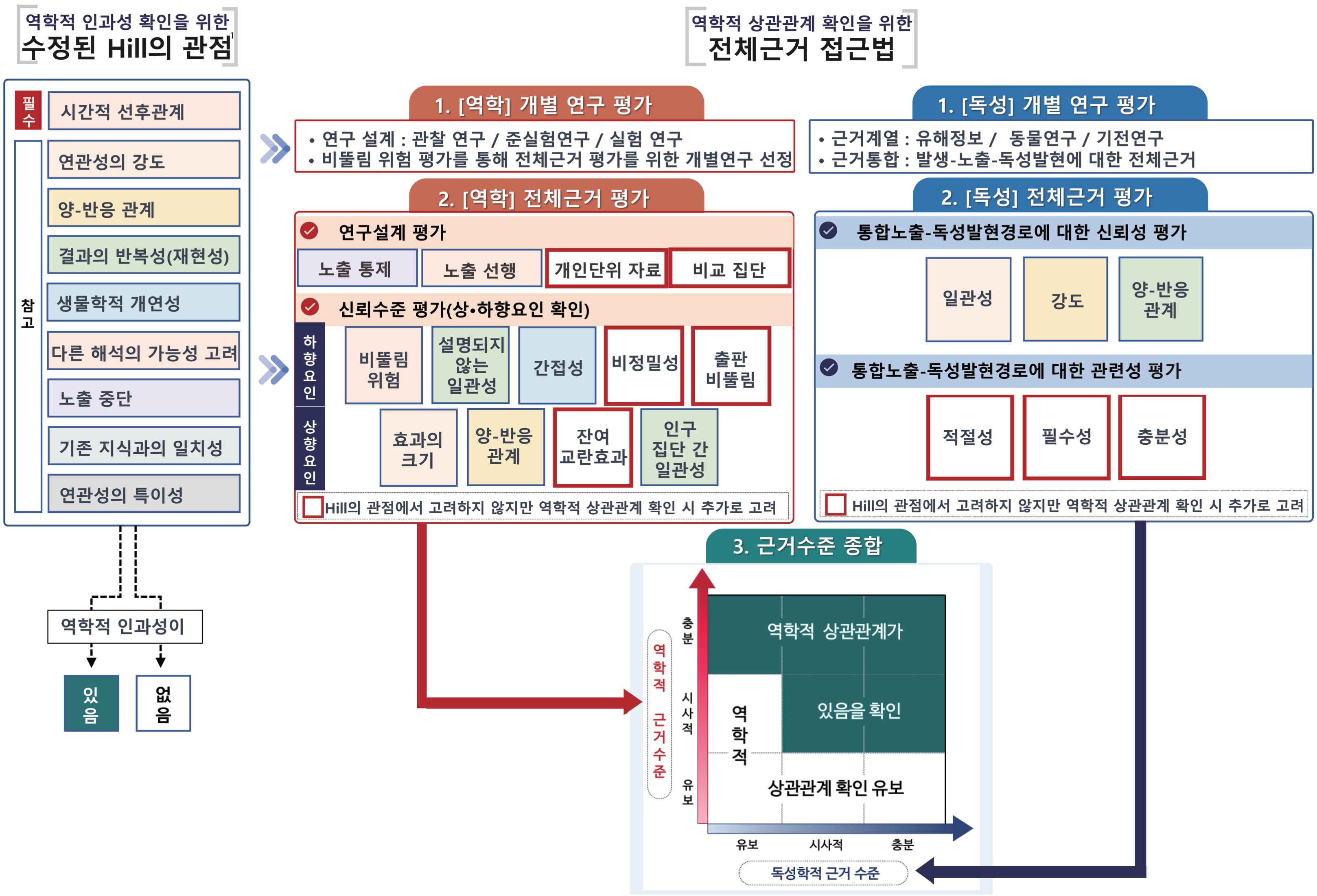

Figure 5. Comparison of weight-of-the-evidence approach with Hill's viewpoints for causal inference. Same colors are the same components in both approaches. AEP, aggregate exposure pathway; AOP, adverse outcome pathway. [1]Source for Hill's revised view-points: US Department of Health, Education and Welfare. 1964.

후 정부 당국에서 실시한 전국 조사에서 가습기살균제폐손상(HDLI)이 단 한 건도 새로 발생하지 않은 것이다[33]. 그러나 질병의 잠복기가 길거나 경과가 비가역적인 경우에는 노출이 중단된 후에도 질병이 발생·지속·악화할 수 있다. 따라서 이 관점도 인과성 추론에서 절대적이지 않으며, 관심 질병의 임상적 특성과 자연사가 반드시 고려되어야 한다.

특히 '연관성의 특이성' 관점은 오히려 제외하는 것이 바람직하기까지 한데, 전술한 바와 같이 한 가지 요인에 의해서만 발생하는 질병은 존재하지 않기 때문이다[30]. 석면 유래 특이성 질환인 악성중피종의 경우에서도 석면은 80%의 원인 점유율을 차지하며 나머지 20%는 석면에 의해 설명될 수 없다[34].

Hill의 인과성 검토를 위한 관점은 60여 년 전의 고전적인 역학 모델에 기반한 것이다. 현재는 과거에는 알지 못했던 노출과 질병 발생까지 경로에서 일어나는 세부적인 사건과 기전에 대해 직접적인 관찰과 측정이 가능해졌다[32]. 관찰된 역학적 연관성에 대한 인과성 검토는, 여러 학문 분야에서 성취한 최신의 지식에 근거한 다학제적인 검토가 되어야 하며, 이때 Hill의 관점들은 단지 참고할 수 있는 목록으로 사용되어야 할 것이다[35].

본 연구의 근거종합 방법은 본 연구가 참고한 기존의 다른 방법들과 몇 가지 점에서 다르다.

첫째, 근거종합 결과 제시하고자 하는 목적과 최종 결론에서 차이가 있다. 본 연구에서 제시한 근거종합의 목적은 '가습기살균제 피해구제를 위한 특별법'의 제5항 인과관계 추정에 필요한 3번째 조건인 노출과 질환 간 역학적 상관관계를 확인하는 것이다. 인과관계에 대한 과학적인 판단은 불확실성을 반영하여 확률(%) 혹은 가능성의 수준(레벨) 등으로 표현할 수 있다. 그러나 원고와 피고 간 다툼에서 어느 일방의 승 혹은 패를 결정해야 하는 법적 판단은 역학적 상관관계가 확인되었는지 여부, 즉 이분적 범주를 요구한다. 다른 한편 OHAT의 경우는 특정 인자의 위해성에 대하여 결정하여 관리의 필요성과 수준을 제시하는 것이 목적이어서 보다 더 세분화된 구분으로 차별적인 수준의 관리정책의 근거를 제시하게 된다. 따라서 본 근거종합의 최종 결론은 OHAT에서 최종 결론으로 유해성을 4가지 수준으로 제시하는 것과는 달리, '역학적 상관관계가 확인' 여부라는 2가지 수준('유보'를 포함한다면 3가지 수준)으로 제시한다.

둘째, 본 근거종합 방법 중 개별 역학 연구의 비뚤림 위험 평가는 기존의 진단 혹은 치료법에 대한 근거종합방법에 비하여 좀 더 관대하다. 예를 들어 ROBINS-I는 사람을 대상으로 하는 임상시험 연구, 혹은 통상적인 치료과정에서 관찰한 코호트 혹은 환자-대조군 연구에 대하여 비뚤림 위험을 평가하는 방법이다[36]. 이러한 개입연구(intervention studies)는 개입(혹은 노출)이 (잠재적) 이득(benefit)이 있을 것이라 가정하고 사람을 대상으로 시험(trial)을 하지만, 본 연구에서 다루는 노출은 (잠재적) 위험(risk)을 가정하므로 사람을 대상으로 하는 무작위/비무작위 배정의 '시험(trial)'이 가능하지 않다. 따라서 검토하게 되는 사람 대상 연구는 대부분 비시험(non-intervention, non-trial), 관찰적 역학연구이다. 관찰연구는 시험연구에 비하여 여러 비뚤림에 취약하여, 시험연구에 적용하는 기준을 그대로 적용하면 대부분의 사람 연구의 결과가 근거종합에서 배제되게 된다. 따라서 직접적인 근거가 되는 사람에서 관찰된 연구결과를 근거종합 시에 최대한 활용하기 위해서는 좀 더 유연한 평가기준을 적용할 필요가 있다[37].

탈리도마이드 약화사건, 고엽제 사건, 9-11 쌍둥이빌딩 폭파사건 등 과거의 심각한 건강피해 사례들은 원인 유해인자에 대한 노출이 중단된 이후에도 수십 년 이상 서로 다른 잠복기를 가지며 다양한 건강피해가 발생한다는 것을 보여준다. 때때로 이들 건강피해는 노출된 피해자들의 자손세대까지 이어지며 지속적인 사회문제가 되었다. 본 연구를 통해 개발한 인과성 추정방법은 가습기살균제 피해 생존자들에 대한 장기 모니터링 등을 통해 향후 축적되는 새로운 지식에 기반하여 개선, 보완되어 나가야 할 것이다.

결 론

본 연구에서 확립한 역학적 상관관계의 확인 방법은, 수정된 Hill의 인과성 검토를 위한 관점을 모두 포괄하고 있을 뿐 아니라, 존재하는 관련 연구의 타당도(질적 수준)를 체계적으로 고려한다.

또한 역학, 독성학 및 임상사례 등 모든 근거들을 종합적으로 고려하므로, 개별 학문분야에서 현저히 발전된 기술과 방법론이 반영될 뿐 아니라, 여러 학문 분야의 과학적 성과가 융합된 결과가 체계적으로 반영된 것이라 할 수 있다.

Hill의 인과성 검토에서 중요한 관점들은 역학적 상관관계의 확인 과정에서 모두 고려되고 있다. Hill의 인과성 검토에서는 각 관점들이 충족되었는지 아닌지의 여부로 평가하는 반면에 역학적 상관관계의 확인 과정에서는, 이러한 관점들이 근거수준에서 차지하는 역할과 위치가 고려된다. 해당 관점들은 전체근거의 신뢰수준 평가 시, 전체근거 신뢰수준의 상·하향 요인 프로파일링 시, 또한 건강영향에 대한 종합 판단(근거수준 종합) 시에 반복적·입체적으로 반영된다.

뿐만 아니라, 역학적 상관관계의 확인은 Hill의 관점에서는 고려되고 있지 않은 연구설계요인 중 자료의 단위(개인단위 vs. 집단단위)와 비교집단 사용여부, 표본의 수가 고려된 비정밀성, 잔여교란효과와 출판비뚤림까지 모두 고려하고 있다.

이와 같이 역학적 상관관계 확인 방법(전체근거 접근법)은 인과성 검토를 위한 다양한 요인을 체계적이고 구체적으로 고려함으로써, Hill의 관점을 활용한 방법이 개별 항목에 대한 구체적인 평가기준을 제시하지 않아 발생할 수 있는 검토자의 주관적 판단 가능성이 줄어든 것으로 볼 수 있다.

본 고에서 저자들은 가습기살균제 노출로 인한 건강피해의 법적 인과관계를 추정하는 데 필요한 역학적 상관관계의 확인방법으로 전체 과학적 근거를 체계적으로 종합하는 방법을 제시하였다. 이는 법적 증거방법에서 개별 연구가 가지는 제한점이나 불확실성을 이유로 배척하지 않고, 전체 증거의 부분으로서 종합적으로 접근하는 것을 통해 사실 관계에 도달하는 것이다. 이것은 향후 인과관계 추정에서 전범(model)이 될 수 있다.

참고문헌

1. Committee on the Investigation of Humidifier Disinfectant Lung Injury. White paper on humidifier disinfectant induced health hazards: from recognition of the outbreak to the establishment of the diagnostic criteria. Seoul: Ministry of Health and Welfare: 2014, p. 37-67 (Korean).
2. Ha M, Jung SH. Introduction to environmental health policy. Yongin: Dankook University Press; 2022, p. 491-505 (Korean).
3. Korea Ministry of Government Legislation. Special Act on Remedy for Damage Caused by Humidifier Disinfectants [cited 2023 Nov 1]. Available from: https://law.go.kr/engLsSc.do?menuId=1&subMenuId=21&tabMenuId=117&query=%EA%B0%80%EC%8A%B5%EA%B8%B0%EC%82%B4%EA%B7%A0%EC%A0%9C#.
4. Korea Ministry of Government Legislation. Enforcement Decree of the Special Act on Remedy for Damage Caused by Humidifier Disinfectants [cited 2023 Nov 1]. Available from: https://www.law.go.kr/%EB%B2%95%EB%A0%B9/%EA%B0%80%EC%8A%B5%EA%B8%B0%EC%82%B4%EA%B7%A0%EC%A0%9C%ED%94%BC%ED%95%B4%EA%B5%AC%EC%A0%9C%EB%A5%BC%EC%9C%84%ED%95%9C%ED%8A%B9%EB%B3%84%EB%B2%95%EC%8B%9C%ED%96%89%EB%A0%B9.
5. National Institute of Environmental Research (NIER). NIER report of review on epidemiological correlations between humidifier disinfectants exposure and health effects; 2022 [cited 2023 Jun 1]. Available from: https://ecolibrary.me.go.kr/nier/#/search/detail/5877095 (Korean).
6. U.S. Department of Health and Human Services. Handbook for conducting a literature-based health assessment using OHAT approach for systematic review and evidence integration; 2019 [cited 2023 Jun 1]. Available from https://ntp.niehs.nih.gov/sites/default/files/ntp/ohat/pubs/handbookmarch2019_508.pdf.
7. Guyatt GH, Oxman AD, Vist G, Kunz R, Brozek J, Alonso-Coello P, et al. GRADE guidelines: 4. Rating the quality of evidence--study limitations (risk of bias). J Clin Epidemiol 2011;64:407-415.
8. Adami HO, Berry SC, Breckenridge CB, Smith LL, Swenberg JA, Trichopoulos D, et al. Toxicology and epidemiology: improving the science with a framework for combining toxicological and epidemiological evidence to establish causal inference. Toxicol Sci 2011;122:223-234.
9. Whaley P, Piggott T, Morgan RL, Hoffmann S, Tsaioun K, Schwingshackl L, et al. Biological plausibility in environmental health systematic reviews: a GRADE concept paper. Environ Int 2022;162:107109.
10. Price PS, Jarabek AM, Burgoon LD. Organizing mechanism-related information on chemical interactions using a framework based on the aggregate exposure and adverse outcome pathways. Environ Int 2020;138:105673.
11. Bian Q, Ping Y, Jun W, Lyu Z, Song Y, Zhang L, et al. A new method to evaluate toxicological data reliability in risk assessments. Toxicol Lett 2019;311:125-132.
12. Tan YM, Leonard JA, Edwards S, Teeguarden J, Paini A, Egeghy P. Aggregate exposure pathways in support of risk assessment. Curr Opin Toxicol 2018;9:8-13.
13. Collier ZA, Gust KA, Gonzalez-Morales B, Gong P, Wilbanks MS, Linkov I, et al. A weight of evidence assessment approach for adverse outcome pathways. Regul Toxicol Pharmacol 2016;75:46-57.
14. Organization for Economic Cooperation and Development (OECD). Users' handbook supplement to the guidance document for developing and assessing adverse outcome pathways: OECD series on adverse outcome pathways No. 1; 2018 [cited 2023 Jun 1]. Available from: https://doi.org/10.1787/5jlv1m9d1g32-en.
15. Supreme Court Decision 72Da1774 Decide December 10, 1974 [cited 2023 Jan 5]. Available from: https://glaw.scourt.go.kr/wsjo/panre/sjo100.do?contId=2076822&q=72%EB%8B%A41774&nq=&w=panre&section=panre_tot&subw=&subsection=&subId=1&csq=&groups=6,7,5,9&category=&outmax=1&msort=&onlycount=&sp=&d1=&d2=&d3=&d4=&d5=&pg=1&p1=&p2=&p3=&p4=&p5=&p6=&p7=&p8=&p9=&p10=&p11=&p12=&sysCd=WSJO&tabGbnCd=&saNo=&joNo=&lawNm=&hanjaYn=N&userSrchHistNo=&poption=&srch=&range=&daewbyn=N&smpryn=N&idgJyul=01&newsimyn=Y&trtyNm=&tabId=&save=Y&bubNm=.
16. Supreme Court Decision 2006Da17539 Decide July 12, 2013 [cited 2023 Jan 5]. Available from: https://glaw.scourt.go.kr/wsjo/panre/sjo100.do?contId=2129559&q=2006%EB%8B%A417539&nq=&w=panre&section=panre_tot&subw=&subsection=&subId=

1&csq=&groups=6,7,5,9&category=&outmax=1&msort=&onlycount=&sp=&d1=&d2=&d3=&d4=&d5=&pg=1&p1=&p2=&p3=&p4=&p5=&p6=&p7=&p8=&p9=&p10=&p11=&p12=&sysCd=WSJO&tabGbnCd=&saNo=&joNo=&lawNm=&hanjaYn=N&userSrchHistNo=&poption=&srch=&range=&daewbyn=N&smpryn=N&idgJyul=01&newsimyn=Y&trtyNm=&tabId=&save=Y&bubNm=.
17. Hill AB. The environment and disease: association or causation? Proc R Soc Med 1965;58:295-300.
18. National Research Council; Federal Judicial Center. Reference manual on scientific evidence; 2011 [cited 2023 Jan 5]. Available from: https://nap.nationalacademies.org/catalog/13163/reference-manual-on-scientific-evidence-third-edition.
19. Lee YG. Proving causation by epidemiologic data in toxic torts. Korean Lawyers Assoc J 2012;61:110-150 (Korean).
20. Supreme Court Decision 2011Da22092 Decide April 10, 2014 [cited 2023 Jan 7]. Available from: https://glaw.scourt.go.kr/wsjo/panre/sjo100.do?contId=2143219&q=2011%EB%8B%A422092&nq=&w=panre&section=panre_tot&subw=&subsection=&subId=1&csq=&groups=6,7,5,9&category=&outmax=1&msort=&onlycount=&sp=&d1=&d2=&d3=&d4=&d5=&pg=1&p1=&p2=&p3=&p4=&p5=&p6=&p7=&p8=&p9=&p10=&p11=&p12=&sysCd=WSJO&tabGbnCd=&saNo=&joNo=&lawNm=&hanjaYn=N&userSrchHistNo=&poption=&srch=&range=&daewbyn=N&smpryn=N&idgJyul=01&newsimyn=Y&trtyNm=&tabId=&save=Y&bubNm=.
21. Seoul High Court Decision 2002Na32662 Decide January 26, 2006 [cited 2023 Jan 7]. Available from: https://glaw.scourt.go.kr/wsjo/panre/sjo100.do?contId=2132454&q=2002%EB%82%9832662&nq=&w=panre&section=panre_tot&subw=&subsection=&subId=1&csq=&groups=6,7,5,9&category=&outmax=1&msort=&onlycount=&sp=&d1=&d2=&d3=&d4=&d5=&pg=1&p1=&p2=&p3=&p4=&p5=&p6=&p7=&p8=&p9=&p10=&p11=&p12=&sysCd=WSJO&tabGbnCd=&saNo=&joNo=&lawNm=&hanjaYn=N&userSrchHistNo=&poption=&srch=&range=&daewbyn=N&smpryn=N&idgJyul=01&newsimyn=Y&trtyNm=&tabId=&save=Y&bubNm=.
22. Environment and Labor Committee, the National Assembly of Korea. Partial amendment review report for Special Act on Remedy for Damage Caused by Humidifier Disinfectants; 2020 [cited 2023 Jan 8]. Available from: https://law.go.kr/lsInfoP.do?lsiSeq=215909&chrClsCd=010203&urlMode=engLsInfoR&viewCls=engLsInfoR#0000 (accessed on Nov 1, 2023) (Korean).
23. National Assembly Secretariat. Minutes of the 376th legislation and judiciary committee meeting: partial amendment for Special Act on Remedy for Damage Caused by Humidifier Disinfectants; 2020 [cited 2023 Jun 8]. Available from: https://likms.assembly.go.kr/record/mhs-10-050.do (Korean).
24. Park TH. The analysis of judicial doctrines concerning proving causation between toxic substances and certain diseases through epidemiological studies in environmental damages lawsuits. Dankook Law Rev 2014;38:173-199 (Korean).
25. Supreme Court Decision 2008Da16776 September 29, 2011 [cited 2023 Jan 10]. Available from: https://glaw.scourt.go.kr/wsjo/panre/sjo100.do?contId=2060398&q=2008%EB%8B%A416776&nq=&w=panre&section=panre_tot&subw=&subsection=&subId=1&csq=&groups=6,7,5,9&category=&outmax=1&msort=&onlycount=&sp=&d1=&d2=&d3=&d4=&d5=&pg=1&p1=&p2=&p3=&p4=&p5=&p6=&p7=&p8=&p9=&p10=&p11=&p12=&sysCd=WSJO&tabGbnCd=&saNo=&joNo=&lawNm=&hanjaYn=N&userSrchHistNo=&poption=&srch=&range=&daewbyn=N&smpryn=N&idgJyul=01&newsimyn=Y&trtyNm=&tabId=&save=Y&bubNm=.
26. Seoul Central District Court Decision 2019Gohap142, 388, 501 Decide January 12, 2021 [cited 2023 Jan 10]. Available from: https://casenote.kr/%EC%84%9C%EC%9A%B8%EC%A4%91%EC%95%99%EC%A7%80%EB%B0%A9%EB%B2%95%EC%9B%90/2019%EA%B3%A0%ED%95%A9142.
27. McGarity TO. Daubert and the proper role for the courts in health, safety, and environmental regulation. Am J Public Health 2005;95 Suppl 1:S92-S98.
28. Krimsky S. The weight of scientific evidence in policy and law. Am J Public Health 2005;95 Suppl 1:S129-S136.
29. EFSA Scientific Committee; Hardy A, Benford D, Halldorsson T, Jeger MJ, Knutsen HK, et al. Guidance on the use of the weight of evidence approach in scientific assessments. EFSA J 2017;15:e04971.
30. Celentano DD, Szklo M. Gordis epidemiology. 6th ed. London: Elsevier; 2018, p. 293-295.
31. Korean Society of Preventive Medicine. Preventive medicine and public health. 4th ed. Seoul: Gyechuk Munwhasa; 2022, p. 60-67 (Korean).
32. Fedak KM, Bernal A, Capshaw ZA, Gross S. Applying the Bradford Hill criteria in the 21st century: how data integration has changed causal inference in molecular epidemiology. Emerg Themes Epidemiol 2015;12:14.

33. Kim KW, Ahn K, Yang HJ, Lee S, Park JD, Kim WK, et al. Humidifier disinfectant-associated children's interstitial lung disease. Am J Respir Crit Care Med 2014;189:48-56.
34. Tossavainen A. Asbestos, asbestosis, and cancer: the Helsinki criteria for diagnosis and attribution. Scand J Work Environ Health 1997;23:311-316.
35. Etzel RA, Grandjean P, Ozonoff DM. Environmental epidemiology in a crossfire. Environ Health 2021;20:91.
36. Sterne JA, Hernán MA, Reeves BC, Savović J, Berkman ND, Viswanathan M, et al. ROBINS-I: a tool for assessing risk of bias in non-randomised studies of interventions. BMJ 2016;355:i4919.
37. Eick SM, Goin DE, Chartres N, Lam J, Woodruff TJ. Assessing risk of bias in human environmental epidemiology studies using three tools: different conclusions from different tools. Syst Rev 2020;9:1-13.
