# Supplementary material for: Evidence integration on health damage for humidifier disinfectant exposure and legal presumption of causation
Source: Epidemiol Health. 2023 Oct 24;45:e2023095. doi: 10.4178/epih.e2023095 (PMC10876420; doi:10.4178/epih.e2023095)
Supplement: Supplementary Material 1. — ey content that needs to be summarized when reviewing selected epidemiological studies [file epih-45-e2023095-Supplementary-1.docx]

Supplementary Material 1. Key content that needs to be summarized when reviewing selected epidemiological studies

| **Category** | **Key Contents** | **Examples of risk of bias *** |
| --- | --- | --- |
| **Funding** | Funding source(s) |  |
|  | Reporting of conflict of interest (COI) by authors | reporting bias |
| **Subjects** | Study population name/description |  |
|  | Dates of study and sampling time frame |  |
|  | Geography (country, region, state, etc.) |  |
|  | Demographics (sex, race/ethnicity, age or lifestage at exposure and at outcome assessment) | missing data bias |
|  | Number of subjects (target, enrolled, n per group in analysis, and participation/follow-up rates) | selection bias |
|  | Inclusion/exclusion criteria/recruitment strategy |  |
| **Methods** | Study design (e.g., prospective or retrospective cohort, nested case-control study, cross-sectional, population-based case-control study, intervention, case report, etc.) |  |
|  | Length of follow-up | information bias |
|  | Health outcome category (e.g., cardiovascular) |  |
|  | Health outcome (e.g., blood pressure) | reporting bias |
|  | Diagnostic or methods used to measure health outcome | information bias |
|  | Confounders or modifying factors and how considered in analysis | confounding bias |
|  | Substance name and CAS number |  |
|  | Exposure assessment (e.g., blood, urine, hair, air, drinking water, job classification, residence, administered treatment in controlled study, etc.) | information bias |
|  | Methodological details for exposure assessment (e.g., HPLC-MS/MS, limit of detection) | information bias |
|  | Statistical methods | information bias |
| **Results** | Exposure levels (e.g., mean, median, measures of variance as presented in paper, such as SD, SEM, 75th/90th/95th percentile, minimum/maximum); range of exposure levels, number of exposed cases |  |
|  | Statistical findings (e.g., adjusted β, standardized mean difference, adjusted odds ratio, standardized mortality ratio, relative risk, etc.) or description of qualitative results |  |
|  | Observations on dose response (e.g., trend analysis, description of whether dose-response shape appears to be monotonic, non-monotonic |  |

* Examples of risk of bias that may be founded when reviewing specific studies

(Source) Reorganize the contents of Handbook of OHAT approach
