# Supplementary material for: Evidence integration on health damage for humidifier disinfectant exposure and legal presumption of causation
Source: Epidemiol Health. 2023 Oct 24;45:e2023095. doi: 10.4178/epih.e2023095 (PMC10876420; doi:10.4178/epih.e2023095)
Supplement: Supplementary Material 2. — Assessment of risk of bias in individual epidemiological studies [file epih-45-e2023095-Supplementary-2.docx]

Supplementary Material 2. Assessment of risk of bias in individual epidemiological studies

| **Types of Bias** | | **Risk of Bias Tool** | **Response Options** | **Risk of Bias Assessment** |
| --- | --- | --- | --- | --- |
| **Key Criteria** | **Detection Bias** | Can we be confident in the exposure characterization? | Risk of Bias Ratings     \| **++** \| Definitely low \| \| --- \| --- \| \| **+** \| Probably low \| \| **-** \| Probably high \| \| **--** \| Definitely high \| | \| **Risk of Bias** \| \| \| \| --- \| --- \| --- \| \| **Tier** \| **Key factor** \| **Other factors** \| \| **1** \| **++/+** \| **Mostly** **++/+** \| \| **2** \| Things that do not meet 1, 3 tiers \| \| \| **3** \| **-/--** \| **Mostly**  **-/--** \| |
|  |  | Can we be confident in the outcome assessment? |  |  |
|  | **Confounding Bias** | Did the study design or analysis account for important confounding and modifying variables? |  |  |
| **Selection Bias** | | Did selection of study participants result in appropriate comparison groups? |  |  |
| **Attrition/Exclusion Bias** | | Were outcome data complete without attrition or exclusion from analysis? |  |  |
| **Selective Reporting Bias** | | Were all measured outcomes reported? |  |  |
| **Other Sources of Bias** | | Were statistical methods appropriate? |  |  |
|  |  | Did researchers adhere to the study protocol? |  |  |

(Source) Reorganize the contents of Handbook of OHAT approach
