# Supplementary material for: Evidence integration on health damage for humidifier disinfectant exposure and legal presumption of causation
Source: Epidemiol Health. 2023 Oct 24;45:e2023095. doi: 10.4178/epih.e2023095 (PMC10876420; doi:10.4178/epih.e2023095)
Supplement: Supplementary Material 3. — Determination of downgrading or upgrading factors in assessment of the confidence level of the cumulative evidence [file epih-45-e2023095-Supplementary-3.docx]

Supplementary Material 3. Determination of downgrading or upgrading factors in assessment of the confidence level of the cumulative evidence

|  | **Factors** | **Determination** |
| --- | --- | --- |
| Downgrading Factors | Risk of Bias | In the result of the perverse risk assessment according to Table 3-1-6 for each preliminarily evaluated study design, there is no downward factor if the first-class study is more than 50 %, and if it is less than 30%, it is lowered by one grade, and if it is less than 30%, it is lowered by two grades are lowered. |
|  | Inconsistency | Investigators should explore explanations for heterogeneity, and if they cannot identify a plausible explanation, the quality of evidence should be downgraded. However, Inconsistency that can be explained would not be eligible for a downgrade. |
|  | Indirectness | If proxy results are used instead of direct exposure factors (proxy indicators) or important results, the level of evidence is lowered by one grade. However, when the exposed population group is different (e.g., general population vs. occupational group), it does not go down. |
|  | Imprecision | If the result estimate is highly uncertain due to a small number of samples and a large confidence interval  when studies include relatively few patients and few events and thus have a wide confidence interval (CI) around the estimate of the effect. In this case, one may judge the quality of the evidence lower  than it otherwise would be considered because of resulting uncertainty about the results.  - If the standard deviation is greater than the average, the 95% confidence interval of OR and RR is 10 or more, the confidence interval of AR is 100 or more, or if the number of study participants is 100 to 200, or the confidence interval of RR passes 0.5 or 1.5 with 1.  - If the number of study participants is less than 100 and the confidence interval of RR passes 0.5 or 1.5, the two grades are lowered. |
|  | Publication Bias | If the results are underestimated or overestimated due to the selective publication of the study  - Publication Bias affects consistency, precision, perverse risk, and directness, so it is sometimes considered to have already been evaluated.  - When early studies that showed relevance have a small number of samples (there is a possibility that irrelevant studies over time will be published, and reproduction studies will also be published).  - Publication bias should be suspected when studies are uniformly small, particularly when sponsored by industries, non-government organizations (NGOs), or authors with conflicts of interest  - The identification of abstracts or other types of grey literature that do not appear as full-length articles within a reasonable time frame (around 3 to 4 years)  Publication bias will be found, and at this time it will be lowered by one grade. |
| Upgrading  Factors | Large magnitude of effect | In general, GRADE raises one grade if RR >2 or <0.5, and two grades if RR >5 or <0.2.  These are made on a project-specific basis based on discussion by the evaluation team and consultation with technical advisors as needed |
|  | Dose response | Raise one grade if there is a linear or nonlinear positive-response relationship. |
|  | Residual Confounding effect | If there are potential confounding variables that have not been measured only in observational studies, the effective results are diluted, and the ineffective results can be interpreted as more effective in practice, so raise them by one grade. |
|  | Consistency | If the results of different population groups and different types of research are consistent, one grade will be raised. |
|  | Other | If the specificity of the association in cases where the effect is rare or unlikely to have multiple, one grade will be raised. |

(Source) Handbook of OHAT[1] and GRADE[2]

* OR (odds ratio), RR (relative risk), AR (absolute risk)
