# Supplementary material for: Evidence integration on health damage for humidifier disinfectant exposure and legal presumption of causation
Source: Epidemiol Health. 2023 Oct 24;45:e2023095. doi: 10.4178/epih.e2023095 (PMC10876420; doi:10.4178/epih.e2023095)
Supplement: Supplementary Material 4. — Comparison of proof method in TCDD-related injury Cases between trial of appeal and trial at the Supreme Court [file epih-45-e2023095-Supplementary-4.docx]

Supplementary Material 4. Comparison of proof method in TCDD-related injury Cases between trial of appeal and trial at the Supreme Court

| Goal of demonstration | Method of demonstration | |
| --- | --- | --- |
|  | The Appeal Court | The Supreme Court |
| General Causation | **Epidemiological causation**  (=①Temporal relationship, ②Dose-response relationship, ③Reversibility, ④Biological plausibility) | (Considerably high level of)  **Epidemiological correlation** |
| Specific Causation | **Epidemiological causation + Exposure + Disease occurred/worsened after exposure**  (As proved considerable plausibility) | (Epidemiological causation + Exposure + Disease occurred/worsened after exposure)  Additional demonstration including the period and extent of exposure, time of onset, health status and life habit before the exposure, history of disease status, and family medical history, etc (As proved plausibility) |
