# Supplementary material for: Evidence integration on health damage for humidifier disinfectant exposure and legal presumption of causation
Source: Epidemiol Health. 2023 Oct 24;45:e2023095. doi: 10.4178/epih.e2023095 (PMC10876420; doi:10.4178/epih.e2023095)
Supplement: Supplementary Material 5. — Comparison of Article 5 of the Special Act on Remedy for damage caused by humidifier disinfectants after the 2nd amendment (March, 2020) [file epih-45-e2023095-Supplementary-5.docx]

Supplementary Material 5. Comparison of Article 5 of the Special Act on Remedy for damage caused by humidifier disinfectants after the 2^nd^ amendment (March, 2020)

| Before amendment | After amendment |
| --- | --- |
| Article 5 (Presumption of Causation) Where it is highly probable to believe that damage to life or health has been caused by a humidifier disinfectant containing toxic chemical substances, the damage to life or health shall be presumed to have been caused by the humidifier disinfectant. | Article 5 (Presumption of Causation) Where the following facts are proved to be true, the damage to life or health shall be presumed to have been caused by a humidifier disinfectant: Provided, That the same shall not apply where the humidifier disinfectant supplier proves that such damage has occurred due to other reasons: <Amended on Mar. 24, 2020>  1. The fact of having been exposed to humidifier disinfectants;  2. The fact that a disease has occurred or the existing disease has worsened after exposure to humidifier disinfectants;  3. The fact that the findings of any survey or research determined by Presidential Decrees confirm the epidemiological correlation between exposure to humidifier disinfectants under subparagraph 1 and diseases under subparagraph 2. |
