# Supplementary material for: Evidence integration on health damage for humidifier disinfectant exposure and legal presumption of causation
Source: Epidemiol Health. 2023 Oct 24;45:e2023095. doi: 10.4178/epih.e2023095 (PMC10876420; doi:10.4178/epih.e2023095)
Supplement: Supplementary Material 6. — Types and evidence levels of epidemiological correlation [file epih-45-e2023095-Supplementary-6.docx]

Supplementary Material 6. Types and evidence levels of epidemiological correlation

|  | **Correlation** | **Association** | **Causation** |
| --- | --- | --- | --- |
| Definition | A special form of association in which two factors are linearly related and independent and dependent variables are not distinguished  A ↔ B | When two factors are related and independent and dependent variables are distinct  A → B | When two factors are related as cause and effect  A → B |
| Representative statistical parameter | Correlation coefficient: γ | Correlation coefficient: γ  Regression coefficient: β  Odds ratio (OR), Relative risk (RR, HR)  Excess risk ratio (ERR) | |
| example | Level of Education and income | Smoking and hypertension | Asbestos and mesothelioma |
| Evidence level | Low |  | High |
