# Supplementary material for: Evidence integration on health damage for humidifier disinfectant exposure and legal presumption of causation
Source: Epidemiol Health. 2023 Oct 24;45:e2023095. doi: 10.4178/epih.e2023095 (PMC10876420; doi:10.4178/epih.e2023095)
Supplement: Supplementary Material 7. — Process of weight of evidence assessment by using a body of evidence approach [1] [file epih-45-e2023095-Supplementary-7.docx]

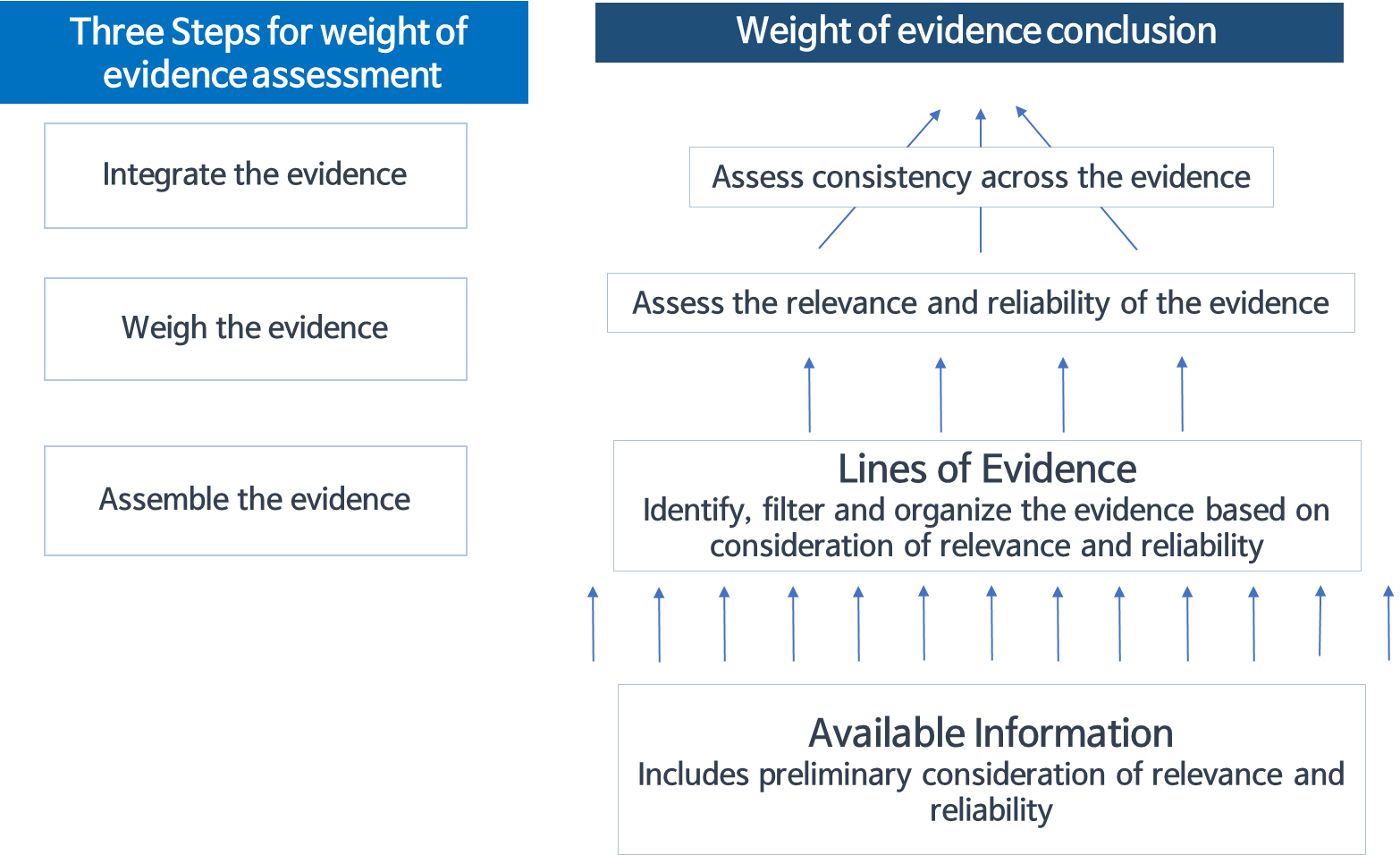


Supplementary Material 7. Process of weight of evidence assessment by using a body of evidence approach [1]
