# Supplementary material for: Evidence integration on health damage for humidifier disinfectant exposure and legal presumption of causation
Source: Epidemiol Health. 2023 Oct 24;45:e2023095. doi: 10.4178/epih.e2023095 (PMC10876420; doi:10.4178/epih.e2023095)
Supplement: Supplementary Material 8. — Comparison of the Hill’s viewpoints with the verification methods of the Epidemiological Correlation [file epih-45-e2023095-Supplementary-8.docx]

Supplementary Material 8. Comparison of the Hill’s viewpoints with the verification methods of the *Epidemiological Correlation*

| **Modified Hill’s viewpoints** | **Verification methods of “Epidemiological Correlation”** |
| --- | --- |
| Temporal relationship | In initial confidence rating for the body of evidence, the confidence level of evidence is given according to the study design, so a design with a clear temporal precedence relationship is given a high grade |
| Strength of the association | Upgrading factor: Upgrade the confidence level of the evidence in consideration of the magnitude of the effect |
| Dose-response relationship | Upgrading factor: Upgrade the confidence level of evidence when there is a dose-response relationship |
| Replication of findings  (Reproducibility) | Upgrading factor: Upgrade the confidence level of evidence when the results are consistent between the target study groups and between the study designs  Downgrading factor: Downgrade the confidence level of evidence when there is unexplained inconsistency |
| Biologic plausibility | Downgrading factor: Downgrade the confidence level of evidence when there is indirectness  In the final assessment of general causality, biological probability is systematically reflected as the level of evidence on health effect is derived and synthesized from the evidences of epidemiology, animal studies, and mechanistic studies. |
| Consideration of alternate explanation | Downgrading factor: Risk of bias attributed to confounding factors is the key assessment factor. Downgrade the confidence level of evidence when the level of risk of bias is low |
| Cessation of exposure | In initial confidence rating for the body of evidence, experimental studies and quasi-experimental studies in which exposure is controlled are given a high grade. |
| Consistency with other knowledge | In the final assessment of general causality, biological probability is systematically reflected as the level of evidence on health effect is derived and synthesized from the evidences of epidemiology, animal studies, and mechanistic studies. |
| Specificity of the association | High grade is given if there is a specificity that explains the biological effects of exposure in biological probability assessment (in animal study). |
